# Multi-omics analysis reveals that ALYREF-mediated m^5^C modification promotes platinum resistance in ovarian cancer via the NSUN2/ALYREF/LGR4 axis

**DOI:** 10.1038/s41419-025-08310-8

**Published:** 2025-12-05

**Authors:** Shimin Yang, Pengyuan He, Wei Wang, Xianming Xu, Xiaowei Xi, Yi Li

**Affiliations:** 1 Department of Obstetrics and Gynecology, Peking University People’s Hospital, Beijing, China; 2 Department of Gynecology and Obstetrics, Shanghai General Hospital, Shanghai Jiao Tong University School of Medicine, Shanghai, China; 3 Department of Gynecologic Oncology, Women’s Hospital, Zhejiang University School of Medicine, Hangzhou, China

**Keywords:** Ovarian cancer, Cancer genomics

## Abstract

Platinum resistance remains a major obstacle to effective treatment and improved prognosis in ovarian cancer. Although 5-methylcytosine (m^5^C) RNA modification has been implicated in chemoresistance, its precise functional role in ovarian cancer remains unclear. In this study, we integrated RNA-Seq and single-cell transcriptomic data from cisplatin-resistant ovarian cancer cell lines and patient samples, identifying the m^5^C reader protein ALYREF as a key regulator of platinum resistance. Functional studies using ALYREF and NSUN2 knockdown, overexpression, and mutant constructs—combined with multi-omics analyses (RNA-Seq, m^5^C-BIS-Seq, and RIP-Seq)—revealed that ALYREF binds to m^5^C-modified LGR4 mRNA, enhancing its stability and promoting activation of the Wnt/β-catenin signaling pathway. Critically, this regulatory mechanism is dependent on NSUN2-mediated m^5^C modification of LGR4 mRNA. Together, our findings demonstrate that the NSUN2/ALYREF/LGR4 axis mediates platinum resistance through m^5^C-dependent stabilization of LGR4 and downstream Wnt signaling activation. Thus, targeting ALYREF may represent a promising strategy to overcome platinum resistance in ovarian cancer.

## Introduction

Ovarian cancer is among the most aggressive gynecological cancers, exhibiting the highest morbidity and mortality rates among female cancers. High-grade serous ovarian cancer (HGSC) is the most prevalent type of epithelial ovarian cancer and the primary cause of mortality in ovarian cancer patients. The majority of ovarian cancer cases are diagnosed at a late stage because subtle or absent early symptoms [1]. Although biomarkers, surgery, genomic testing, chemotherapy, and target therapies are widely used for diagnosing and treating ovarian cancer, nearly all patients experience recurrence or develop resistance to chemotherapy following multiple treatment cycles [2, 3]. Thus, chemotherapy resistance is a significant contributor to recurrence and mortality among ovarian cancer patients, highlighting the clinical importance of in-depth research on the mechanisms of drug resistance to improve therapeutic efficacy.

Building on previous research, we examined the regulatory relationships among key genetic, epigenetic, and transcriptomic factors in ovarian cancer using optimized single-cell multi-omics sequencing technology [4]. Currently, we are conducting follow-up with our patient cohort. Patients are classified into platinum-sensitive and platinum-resistant groups according to their response to platinum-based treatments and recurrence status. This categorization has allowed us to initiate investigations into the mechanisms underlying platinum resistance in ovarian cancer. Combined with RNA-Seq sequencing data on cisplatin-resistant ovarian cancer cells, we discovered that the methyl-binding protein ALYREF is crucial for the emergence of cisplatin resistance in ovarian cancer.

A significant post-transcriptional modification, RNA methylation regulates the output of nuclear RNAs, enhances mRNA stability, and governs RNA-protein interactions [5]. Altered RNA methylation levels are linked to tumors, as well as cardiovascular and neurological diseases [6–8]. The m^5^C methylation modification specifically means the methylation of the RNA’s fifth carbon of cytosine. Xu et al. hypothesized that m^5^C modification may be associated with sensitivity to chemotherapeutic drugs and could impact the current status of ovarian cancer treatment. However, no study has yet provided supporting evidence [9]. ALYREF (Aly/REF export factor), as an intranuclear m^5^C-reading protein, specifically recognizes m^5^C modifications on RNAs through its K171 structural domain, and synergizes with the methyltransferase NSUN2 to regulate the nuclear export and function of target mRNAs. Wang et al. found that ALYREF can promote the splicing and regulate the stability of RABL6/TK1 mRNA in conjunction with NSUN2, thereby facilitating the progression of bladder cancer [10]. Additionally, Nulali, Jin et al. demonstrated that ALYREF regulates mRNA stability and promotes tumor development [11, 12]. ALYREF functions as a specific mRNA m^5^C-binding protein that enhances the nuclear export capacity of mRNAs while simultaneously improving the stability of target mRNAs, ensuring their export to the cytoplasm for translation [13]. Despite the limited studies on ALYREF in tumors, Zhao, Zheng et al. reported that ALYREF may serve as a marker for assessing tumorigenesis and therapeutic efficacy [14], and could be incorporated into prognostic models for ovarian cancer [15]. However, no research has yet examined the contribution of abnormal ALYREF expression to the development and progression of ovarian cancer, as well as its impact on platinum resistance.

In the canonical Wnt/β-catenin signaling pathway, the Wnt protein binds to the Frizzled (FZD) receptor and the low-density lipoprotein receptor-related protein 5/6 (LRP5/6) co-receptor, forming a ternary complex that inhibits the β-catenin destruction complex (CK1α, APC, GSK3β, AXIN), stabilizing cytoplasmic β-catenin. The accumulated β-catenin then moves to the nucleus, where it joins forces with TCF/LEF transcription factors to stimulate the expression of specific genes (e.g., MYC, CCND1 and AXIN2), driving cell proliferation, stemness, and survival. In contrast, the non-canonical Wnt pathway regulates cell polarity and calcium signaling independently of β-catenin. Dysregulated canonical Wnt signaling, marked by β-catenin stabilization, is a hallmark of ovarian cancer and other malignancies.

In ovarian cancer, aberrant Wnt signaling drives platinum resistance [16]. Platinum-resistant HGSC cell lines and PDX models show elevated β-catenin levels and heightened transcriptional activity of downstream targets (TCF7, FZD1, LEF-1) [17]. Mechanistically, Wnt activation promotes chemoresistance by upregulating CSC markers to enrich drug-resistant subpopulations and inducing EMT and enhancing drug efflux [18]. Preclinically, β-catenin inhibitors reverse cisplatin resistance, confirming the pathway’s centrality in platinum tolerance [19]. LGR4, a Wnt co-receptor complexed with FZD and R-spondin, amplifies β-catenin signaling to sustain pro-survival gene networks and CSC self-renewal. In colorectal cancer, LGR4 overexpression upregulates the ferroptosis inhibitor SLC7A11 via Wnt-dependent mechanisms, fostering chemoresistance, while LGR4 blockade induces lipid peroxidation to restore drug sensitivity [20]. Though understudied in ovarian cancer, LGR4 may similarly regulate platinum resistance.

In our study, we found that NSUN2 regulates the activity of the Wnt/β-catenin signaling pathway by catalyzing m^5^C modification within the coding sequence (CDS) of LGR4 mRNA, a modification that is subsequently recognized by ALYREF. This interaction enhances the stability and nuclear export of LGR4 mRNA, thereby contributing to platinum resistance in ovarian cancer.

## Materials and methods

### Clinical sample

The acquisition of single-cell samples has been thoroughly documented in our previous research [4]. We will categorize the patients based on the following criteria. Platinum-sensitive type: patients who achieve clinical remission or experience recurrence after discontinuing platinum-based chemotherapy for more than 6 months. Platinum-resistant type: patients who have not responded effectively to chemotherapy, including those whose tumors persisted or progressed during initial treatment, and those who relapsed within 6 months after discontinuing chemotherapy. In this study, a total of 67 HGSC tissues and 13 fallopian tube (FT) tissues were obtained from Shanghai General Hospital. Patients were informed about the operations and asked to provide informed consent prior to the procedures. Ethical approval was granted by the Shanghai General Hospital and Peking University People’s Hospital (2022PHB375-001). All methods were performed in accordance with the relevant guidelines and regulations.

### Data resources

A total of 381 files containing clinical information and expressed sequence tag data in fragments per kilobase of transcript per million mapped fragments format were obtained from The Cancer Genome Atlas (TCGA). SOFT expression matrix data, containing GSE33482, GSE45553 [21], GSE98559 [22] and GSE140996, were acquired from the Gene Expression Omnibus (GEO) database (https://www.ncbi.nlm.nih.gov/geo/).

### Immunohistochemistry (IHC)

The tissue microarrays were initially placed in an incubator (DNP-9052, Jinghong, Shanghai) at 60 °C for 24 h to melt the surface sealing wax. The tissue microarrays were then immersed in xylene and a series of ethanol solutions of varying concentrations in a hood to remove the paraffin wax and rehydrate the tissue. Antigen retrieval was performed in boiling water for 7 min using citrate antigen retrieval solution (P0081; Beyotime, China). To get rid of endogenous peroxidase activity, tissue sections were treated with 3% hydrogen peroxide, followed by blocking with 10% goat serum (C0265; Beyotime, China) for 1 h at room temperature. Following a rinse with PBS, the tissue microarrays were incubated at 4 °C for 16 h with anti-ALYREF antibody (16690-1-AP; Proteintech, China) and anti-LGR4 antibody (20150-1-AP; Proteintech, China), each at a 1:300 dilution. The Secondary antibody (GB23303; Servicebio, China) and DAB chromogen (G1212; Servicebio, China) were utilized for secondary antibody incubation and staining of the tissue microarray. The tissue microarrays were stained with haematoxylin (G1004; Servicebio, China) for 3 min, after which the staining was terminated in distilled water. The tissue microarrays were immersed in xylene and gradients of ethanol concentrations for dehydration. Finally, the sections were sealed with neutral resin (G1404; Servicebio, China). Images were obtained using a microscope (Leica, London, UK). Image analysis was performed using ImageJ software (version 1.52a; National Institutes of Health) [23] as follows:

Cytoplasmic staining scores were calculated using the following sequence: Plugins—IHC Profiler—Cytoplasmic Stained Image—H DAB. Nuclear staining scores were calculated via Plugins—IHC Profiler—Nuclear Stained Image—H DAB—Set Threshold, followed by Plugins—Macros—IHC Profiler calculation. To determine the percentage of positive cells, the image type was converted to RGB Stack via Image—Type—RGB Stack, and the progress bar was adjusted to the center. Thresholding was performed using Image—Adjust— Threshold, and cell positivity was quantified by Analyze—Set Measurements with Area, Mean Gray Value, and Area Fraction selected, followed by the Measure function.

The standard scoring system is as follows: Negative: 0 points; Weakly positive: 1 point; Positive: 2 points; Strongly positive: 3 points.

4 grades based on the percentage of positive cells scored: 1 point for 0%≤ percentage of positive cells ≤25%; 2 points for 25% < percentage of positive cells ≤50%; 3 points for 50% < percentage of positive cells ≤ 75%; 4 points for 75%<percentage of positive cells ≤100%.

Cell staining intensity score = cytoplasmic staining score + nuclear staining score

IHC score = Percentage of positive cells score * Cell staining intensity score.

### Cell culture and cell growth

A2780, SKOV3 and 293 T cells were obtained from the National Collection of Authenticated Cell Cultures (Shanghai, China). All cell lines were authenticated by STR profiling and routinely tested to confirm they were free of mycoplasma contamination. SKOV3 and 293 T cells were cultured in high-glucose Dulbecco’s Modified Eagle Medium (DMEM; 41401ES76, Yeasen, China) supplemented with 10% fetal bovine serum (40131ES76, Yeasen, China). A2780 cells were maintained in RPMI-1640 medium (41402ES76, Yeasen, China) with 10% fetal bovine serum. All cell lines were cultured under standard incubator conditions of 37 °C and 5% CO₂ using an incubator (51023126; Thermo Fisher Scientific, USA).

### Cisplatin-resistant cell line construction

A2780 and SKOV3 cells in the logarithmic growth phase were cultured in 96-well plates at a density of 10⁵ cells per well. The half-maximal inhibitory concentration (IC50) of the drugs was then determined following an overnight incubation period. Once the cell growth density reached 80–90%, the cells were treated by adding cisplatin at a starting concentration of one-fifth of the IC50 of the parental cell line, after which they were cultured continuously. When the cell density reached approximately 50%, the culture medium was discarded, and the cells were washed with PBS before being replaced with a cisplatin-free medium. Once the cell growth density returned to 80–90%, the previous drug treatment was repeated. Once the cells had stabilized at this concentration, the cisplatin concentration was gradually increased, and the cells were treated similarly until they could grow stably at the final cisplatin concentration, thereby establishing cisplatin-resistant cell lines. Finally, the IC50 of the drug-resistant cell lines was determined. This was determined using the CCK-8 assay. The cisplatin-resistant cell lines are designated A2780/DDP and SKOV3/DDP.

### Plasmid construction and transfection

The plasmids used in this study were obtained from QEgene (Shanghai, China) and verified by DNA sequencing. To produce the shALYREF plasmid, shRNA primer pairs that target ALYREF were obtained from the shRNA library (TRC) and subsequently inserted into the pLKO.1-Puro vector. The ALYREF open reading frame (ORF) was amplified and cloned into the pLV3-CMV vector to construct the ALYREF overexpression plasmid. Similarly, the LGR4 overexpression plasmid was created by amplifying the LGR4 ORF and cloning it into the pLV3-CMV vector. The ALYREF mutant plasmid was constructed based on the observation that the m^5^C binding site of ALYREF is K171 [10]. Mutations were introduced by changing AAG to GCG and K to A, followed by cloning into the pLV3-CMV vector. Cells transfected with the ALYREF knockdown plasmid were designated as shALYREF-1 and shALYREF-2, whereas cells transfected with the control plasmid were designated as shNC. Overexpressing cells were labeled as ALYREF and LGR4, while control plasmid-transfected cells were labeled as NC. The ALYREF mutant plasmid was labeled as ALYREF-mut. The 293 T cells were cultivated in 6 cm dishes until the density of the cells reached 50%. Transfection was then carried out using LipoFiter 3.0 reagent (L3000015; Thermo Fisher Scientific, USA). The 293 T cells were then left to incubate for 48 h. The next step was to collect and filter the sample to separate the lentiviral solution. This solution was added to A2780 and SKOV3 cells seeded in 6-well plates at approximately 40% confluency, using varying concentrations. After 48 h of incubation, the medium was replaced with DMEM (4 mL) supplemented with puromycin (2 μg/mL) to initiate selection. After an additional 24–48 h, the cells exhibiting the highest viability were selected for expansion. Subsequently, the cells were validated using Western blot assay.

### Western blot and antibody

Protein extraction was carried out using RIPA lysis buffer (P0013C; Beyotime, China), which was then supplemented with 1% phenylmethanesulfonyl fluoride (ST507-10ml; Beyotime, China). The protein extracts were then denatured at 95 °C (37 °C for membrane proteins). PAGE gels (PG222; Epizyme Biotech, China) were prepared according to separate protein extracts, which were subsequently transferred to PVDF membranes (ISEQ00010; Merck, USA) following methanol activation. After transfer, the membranes were incubated in protein-free rapid blocking solution (PS108; Epizyme Biotech, China) for 15 min at room temperature. Membranes were then rinsed three times with 0.1% Tris-HCl containing Tween-20 (TBST) for 10 min each. Primary antibodies were applied and incubated overnight at 4 °C, followed by three washes with TBST. The membranes were subsequently incubated with the appropriate secondary antibody at room temperature for 2 h, followed by three additional rinses. Finally, detection was carried out using a chemiluminescence imaging system (ChemiDox, Bio-Rad, USA and Tanon 5200; Tanon, China) and a luminescence kit (SQ201; Epizyme Biotech, China). The antibodies employed in these experiments and their respective dilutions are detailed below:

Anti-ALYREF antibody (16690-1-AP; Proteintech, China) was diluted to 1:1000.

Anti-LGR4 antibody (A12657; ABclonal, China) was diluted to 1:2000.

Anti-β-Catenin antibody (51067-2-AP; Proteintech, China) was diluted to 1:5000.

Anti-c-MYC antibody (10828-1-AP; Proteintech, China) was diluted to 1:2000.

Anti-cyclin D1 antibody (26939-1-AP; Proteintech, China) was diluted to 1:5000.

Anti-GAPDH antibody (60004-1-lg; Proteintech, China) diluted to 1:5000.

Anti-Tubulin antibody (10094-1-AP; Proteintech, China) diluted to 1:2000.

Anti-Rabbit antibody (SA00001-2; Proteintech, China), diluted to 1:2000.

Anti-Mouse antibody (SA00001-1; Proteintech, China) diluted to 1:2000.

Anti-LaminB1 antibody (66095-1-lg; Proteintech, China) diluted to 1:5000

Relative protein levels were compared quantitatively using ImageJ software (version 1.52a; National Institutes of Health, USA) [23].

### RNA extraction and qPCR

Total RNA was extracted using the RNA Extraction Reagent (19201ES60; Yeasen, China). Subsequently, cDNA synthesis was carried out using the Reverse Transcription Reagent (R202-02; EnzyArtisan, China), followed by quantitative PCR (qPCR) analysis using a real-time fluorescence PCR system (QuantStudio 7 Flex; Thermo Fisher Scientific, USA). Normalization of the mRNA expression levels to GAPDH and quantification using the 2^-ΔΔCt^ method [24] was also performed. The primer sequences for qPCR can be found in Table S1.

### Cell proliferation assay

We seeded the cells into 96-well plates at a density of 2,000 cells per well and incubated them for 0, 24, 48, and 72 h. The Cell Counting Kit-8 (C0038; Beyotime, China) was used to assess cell proliferation capacity. After a further hour of incubation, the optical density (OD) was measured at 450 nm using a multimode microplate reader (VLBLATGD2; Thermo Fisher Scientific, USA). The blank control group consisted of DMEM, which was supplemented with 10% FBS.

### Clonogenic assays

For standard clonogenic assays, cells were plated at 800 cells/well in 6-well plates and cultured for 14 days. For drug-gradient clonogenic assays, cells were plated at 1600 cells/well and cultured for 7 days to avoid complete cytotoxicity at higher drug concentrations. The cells were fixed in 4% paraformaldehyde (P0099; Beyotime, China) for 30 min, until either the incubation time reached 14 days or the majority of the clones contained more than 50 cells. Cells were then stained with crystal violet solution (C0121; Beyotime, China) for 30 min.

### RNA dot blot assay

The RNA was extracted as previously described, and its concentration was subsequently calculated. The RNA was incubated at 95 °C for 5 min to denature its secondary structure. The RNA was adjusted to varying concentrations, and 2 μL of RNA per well was applied to a nylon membrane (10600001; GE WHATMAN, UK). Following 1 h of hot cross-linking in a 60 °C oven and 30 min of cross-linking under 254 nm UV light, the membrane was treated with T-TBS (containing 5% skimmed milk powder or BSA). The membrane was subsequently incubated with a 1:2500 dilution of an anti-m^5^C primary antibody (68301-1-Ig; Proteintech, China), followed by incubation with the corresponding secondary antibody. The membranes were then detected using a luminescence kit (SQ201; Epizyme Biotech, China). Subsequently, the membranes were treated with methylene blue dye for 30 min and then washed with TBST.

### Transwell cell migration and invasion assays

For the cell migration assay, 200 μL of serum-free medium containing 1 × 10^5^ SKOV3 cells (2 × 10^5^ A2780 cells) was added to the upper chamber (14421030; Corning Incorporated, USA). Subsequently, 700 μL of DMEM medium containing 10% FBS was added to the lower chamber and incubated for 8 h (24 h for A2780 cells). After incubation, the cells were fixed with 4% paraformaldehyde for 30 min and stained with a crystal violet dye solution for 30 min.

In the cell invasion assay, 60 μL of Matrigel (356234; Corning Incorporated, USA), diluted 1:6 with serum-free medium, was placed in the upper chamber and incubated for 1 h at 37 °C to allow solidification. Cells and culture medium were subsequently placed in the upper chamber following the same protocol used for the cell migration assay. Ultimately, the cells were fixed and stained after 32 h of incubation (48 h for A2780 cells).

### RNA-seq

RNA was extracted from stable ALYREF knockdown A2780 cells and control cells using TRIzol (15596018; Thermo Fisher Scientific, USA) following the provided protocol. The experimental procedure is briefly described as follows: the quantity and purity of total RNA were assessed using a NanoDrop ND-1000 (NanoDrop Wilmington, USA), and RNA integrity was evaluated with a Bioanalyzer 2100 (Agilent, CA, USA). Acceptable criteria for downstream assays included RNA concentrations >50 ng/μL, RIN values > 7.0, and total RNA > 1 μg. Two purification steps were performed using oligo (dT) magnetic beads (25-61005; Thermo Fisher Scientific, USA) to specifically capture mRNA containing polyadenylated (PolyA) sequences. The isolated mRNA was fragmented with the Magnesium Ion Interruption Kit (cat. no. E6150S, USA) at 94 °C for 5 min. Fragmented RNA was reverse transcribed into cDNA using reverse transcriptase (cat. 1896649, California, USA), followed by second-strand synthesis to generate double-stranded DNA. Adapter ligation was then performed. The resulting DNA fragments were size-selected and purified using magnetic beads. The fragments were pre-denatured by PCR to achieve sizes of approximately 300 bp ± 50 bp. Finally, double-stranded sequencing was performed using a HiSeq 4000 system (Illumina, USA) following standard practices. The final data were processed to create high-quality sequencing data (Clean Data), which was then used to compare it with the genomes of Homo sapiens. Each sample consisted of three biological replicates.

### m^5^C-BIS-Seq

m^5^C-BIS-seq was performed by Cloudseq Biotech Inc. (Shanghai, China). In brief, the GenSeq® rRNA Removal Kit (GenSeq Inc., China) was used to remove rRNA from total RNA. The rRNA-depleted samples were then subjected to bisulfite conversion and purification using the EZ RNA Methylation Kit (R5001; Zymo Research, China). The purified RNA was subsequently used for library preparation with the GenSeq® Low Input RNA Library Prep Kit (GenSeq Inc., China), following the manufacturer’s protocol. Quality of the library was assessed with a Bioanalyzer 2100 (Agilent Technologies, China) before sequencing.

### RIP-Seq

RIP-Seq was performed by Cloudseq Biotech Inc. (Shanghai, China). RNA immunoprecipitation (RIP) assays were performed using the GenSeq RIP Kit (GenSeq Inc.), following the manufacturer’s instructions. Briefly, cell lysates were immunoprecipitated with either target protein-specific or IgG control antibodies using protein A/G magnetic beads. After proteinase K digestion, RNA bound to the immunoprecipitated protein was extracted using phenol/chloroform and precipitated with ethanol. The purified RNA was resuspended in RNase-free water, and rRNA was removed using the GenSeq rRNA Removal Kit (GenSeq Inc., China). The rRNA-depleted RNA was then used for library preparation with the GenSeq Low-Input RNA Library Prep Kit (GenSeq Inc., China), following the manufacturer’s protocol. Quality and quantification of the library were assessed using the BioAnalyzer 2100 system (Agilent Technologies, China), following 150 bp paired-end sequencing.

### Bioinformatics analysis

The Seurat R package (version 5.1.0) [25] was used for downsampling and visualization of single-cell sequencing data, while the CellMarker2.0 (http://bio-bigdata.hrbmu.edu.cn/CellMarker/) [26] was used to annotate single-cell clusters. Cell-to-cell communication analysis and visualization were performed using the CellChat R package (version 1.6.1) [27]. Differentially expressed genes (DEGs) were analyzed using the limma R package (version 3.52.4) [28]. Subsequent screening of DEGs was conducted, followed by Kyoto Encyclopedia of Genes and Genomes (KEGG) and Gene Ontology (GO) enrichment analyses using the Gene Set Variation Analysis (GSVA) R package (version 1.52.3). The tagged gene sets for GO and KEGG were obtained from the MsigDB database (http://www.gsea-msigdb.org/gsea/msigdb). KEGG and GO analyses were functionally annotated using the ClusterProfiler R package (version 4.12.6) [29], and gene set enrichment analysis (GSEA) was conducted with the GSEABase R package (version 1.66.0). Finally, the results of the KEGG and GO analyses were visualized using the ggplot2 R package (version 3.5.1) [30]. Drug sensitivity analyses were conducted using the oncoPredict R package (version 1.2) [31] and the pRRophetic R package (version 0.5) [32]. All bioinformatics analyses were conducted using R software (version 4.4.1; https://www.r-project.org/). Correlation analysis between ALYREF and LGR4 mRNA expression was performed using the cBioPortal online platform (https://www.cbioportal.org/), based on HGSC transcriptomic data from the GDC database (July 2024 release).

### EdU assay

EdU assay was carried out using the EdU kit (C0078S; Beyotime, China) according to the provided protocol. Briefly, cells in the logarithmic growth phase were inoculated into 96-well plates and cultured overnight. The EdU working solution was added and incubated for 2 h. The cells were then fixed, washed and permeabilized. The Click Additive Solution was then added, prepared according to the protocol, and the cells were incubated for 30 min in the dark. After washing, the nuclei were stained with Hoechst 33342 and incubated for 10 min at room temperature in the dark. Finally, the cells were visualized under a fluorescence microscope.

### m^5^C RNA RIP assay

The m^5^C-modified regions of the transcriptome were specifically enriched using the GenSeq@m^5^C MeRIP kit (GS-ET-003; Cloud-seq, China) following the provided instructions. In summary, RNA was initially extracted by the aforementioned method, followed by fragmentation into RNA fragments of approximately 200 nucleotides in size. The pre-blocked, antibody-conjugated magnetic beads were then used to incubate the fragmented RNA for 1 h. Finally, the levels of m^5^C-enriched fragment RNA and input RNA were quantified by qRT-PCR. The MeRIP- primers for LGR4 were designed based on the identified methylation sites.

### Tumor xenograft

Female BALB/c nude mice aged 4–6 weeks were obtained from the Chedun Laboratory Animal Breeding Farm Co. The mice were randomly allocated into three experimental groups (seven mice per group) using a random number generator. shALYREF and shNC SKOV3 cells (1 × 10^7^ cells in 100 μL PBS) were injected into the axilla of the mice. Tumor tissue was collected from two groups when the tumors reached a volume of 200 mm^3^ or a diameter exceeding 20 mm. When the tumors in the other two groups reach 100 mm^3^, cisplatin will be injected intraperitoneally for 4 weeks, after which tumor volume, weight, and mouse body weight will be assessed. All procedures were approved by the Chedun Laboratory Animal Ethics Committee (Ethics Code: AD2024124). No blinding was performed in this study. Investigators were aware of group allocations during experiments and outcome assessment.

### Extraction of nuclear and cytoplasmic lysates

Cytoplasmic and nuclear proteins were extracted using the Protein Extraction Kit (P0027; Beyotime, China) to assess the cellular localization of β-catenin. Briefly, adherent cells were incubated in an EDTA solution before being collected by aspiration. The extraction of cytoplasmic proteins was achieved by adding the cytoplasmic protein extraction reagent, followed by incubation on ice for 15 min and centrifuging at 16,000 × *g* for 5 min. Subsequently, nuclear extraction reagent was applied, and after 30 min of periodic vigorous vortexing, nuclear proteins were collected by centrifugation at 16,000 × *g* for 10 min. The purity of cytoplasmic and nuclear proteins was confirmed by Western blot analysis.

### Immunofluorescence

Cells were initially seeded in confocal Petri dishes (WG801001; Servicebio, China). Subsequently, the cells were fixated with 4% paraformaldehyde and washed three times with PBS for 3 min each. Following this, the cells were incubated with 0.5% Triton X-100 (P1080; Solarbio, China) for 20 min at room temperature, followed by blocking with goat serum for 30 min at room temperature after washing with PBS. The cells were then incubated overnight at 4 °C with anti-β-catenin antibody (51067-2-AP; Proteintech, China), diluted at 1:300, and anti-LGR4 antibody (A12657; ABclonal, China), diluted at 1:200. On the following day, after washing with PBS, the diluted Anti-rabbit secondary antibody (4412S; Cell Signaling, USA), at a dilution of 1:1000, was added and incubated for 1 h at room temperature in the dark. Then, the cells were washed with PBS. Following this, DAPI (C1005; Beyotime, China) was added to stain the cell nuclei for 5 min in the dark. Finally, after washing with PBS, a blocking solution containing an Anti-Quench Sealer (P0126; Beyotime, China) was added, and the cells were imaged using a confocal microscope (TCS SP8; Leica, GER).

### RNA nucleolar–cytoplasmic separation experiment

Cytoplasmic and nuclear RNA were extracted separately using a Cytoplasmic and Nuclear RNA Extraction Kit (21000; AmyJet Scientific, China). Briefly, cell suspensions were pelleted by centrifugation and lysed with Lysis Buffer for 15 s, followed by centrifugation at 14,000 × *g* for 10 min. The supernatant was used for cytoplasmic RNA extraction, while the pellet was used for nuclear RNA extraction. Each fraction was mixed with SK Buffer and anhydrous ethanol and then transferred to spin columns for binding. The columns were washed three times with Wash Solution, and RNA was subsequently eluted with DEPC-treated water. Finally, cytoplasmic and nuclear RNA fractions were obtained and analyzed by qPCR.

### Statistics

All experiments were performed in triplicate. Data are presented as mean ± standard deviation (SD). The normality of data distribution was assessed using the Shapiro–Wilk test. Unpaired two-tailed Student’s t-tests were applied to normally distributed data, while the Mann-Whitney U test was used for non-normally distributed data. The assumption of equal variance between groups was assessed using Levene’s test; when variances were unequal, Welch’s t-test was used. Statistical analyses were conducted with SPSS software (version 19.0). Graphs were generated with GraphPad Prism software (version 9.3). A *p*-value less than 0.05 was considered statistically significant. (*: *p* < 0.05, **: *p* < 0.01, ***: *p* < 0.001, ****: *p* < 0.0001).

## Results

### The expression of the m⁵C methylation-binding protein ALYREF is associated with cisplatin resistance in ovarian cancer

**Fig. 1:**
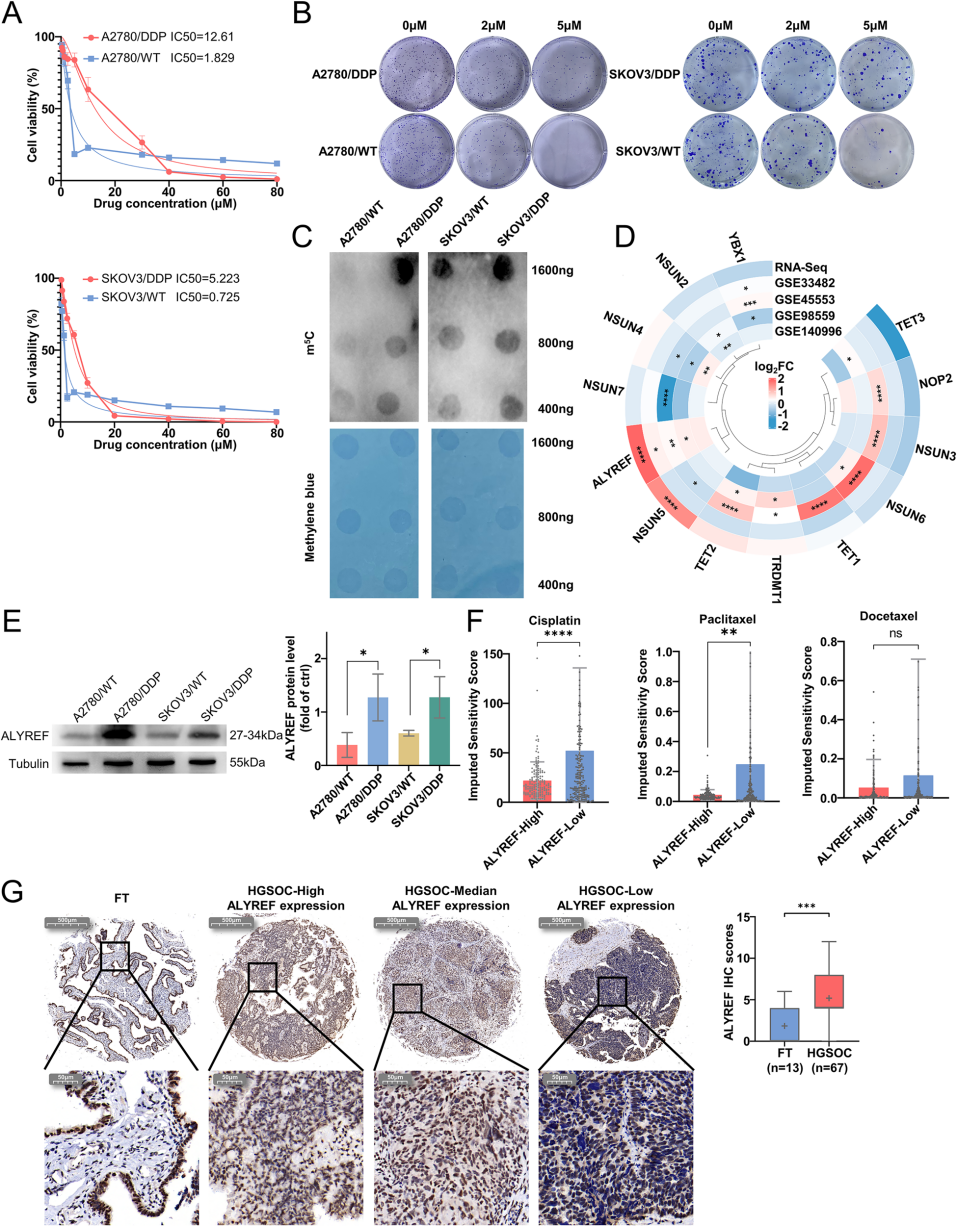
ALYREF is associated with cisplatin resistance in ovarian cancer. **A** CCK-8 assay measuring cisplatin resistance in A2780/DDP and SKOV3/DDP cells. **B** Clone-forming ability of cisplatin-resistant and parental ovarian cancer cells following treatment with varying concentrations of cisplatin. **C** Dot blotting assay detecting overall m^5^C methylation levels in A2780/DDP, SKOV3/DDP and parental cells. **D** Heatmap illustrating the differential expression of m^5^C-related genes in A2780/DDP and parental cells, based on RNA-Seq data and GEO datasets (GSE33482, GSE45553, GSE98559, and GSE140996). **E** Western Blot assay of ALYREF in A2780/DDP, SKOV3/DDP and parental cells. **F** Drug sensitivity analysis based on TCGA data. **G** Representative figures of IHC staining showing ALYREF expression levels in tissue microarray samples. Data are displayed as mean ± SD; *: *p* < 0.05. **: *p* < 0.01. ***: *p* < 0.001. ****: *p* < 0.0001.

Previous studies have demonstrated the pivotal role of the methyltransferase NOP2 in HGSC [33], and Xu et al. have reported a correlation between m⁵C RNA methylation and platinum resistance in ovarian cancer [9]. We therefore focused on investigating the role of m^5^C methylation in platinum resistance in ovarian cancer. We initially constructed cisplatin-resistant A2780 and SKOV3 cell lines to evaluate their resistance (Fig. 1A, B). And we found elevated levels of total RNA m^5^C methylation in A2780/DDP and SKOV3/DDP (Fig. 1C). We sequenced A2780/DDP and parental cell lines by RNA-Seq. From RNA-Seq analysis, we identified 13 genes associated with m⁵C methylation, among which ALYREF, an m⁵C reader, was significantly upregulated. This finding was validated by GSE33482, GSE45553, GSE98559 and GSE140996 data, all of which demonstrated elevated ALYREF expression in cisplatin-resistant ovarian cancer cells (Fig. 1D). Subsequently, significantly elevated levels of ALYREF protein expression in A2780/DDP and SKOV3/DDP were confirmed by western blotting experiments (Fig. 1E). Next, 381 HGSC tissue samples from the TCGA database were divided into ALYREF high- and low-expression groups according to the ALYREF expression levels. Drug sensitivity analysis indicated that the ALYREF high-expression group had reduced sensitivity to cisplatin and paclitaxel (Fig. 1F). Additionally, ALYREF expression was found to be elevated in HGSC tissues by immunohistochemistry in 67 HGSC tissues and 13 FT tissues (Figs. 1G and S1A). An analysis of follow-up data from 61 HGSC patients revealed that ALYREF was associated with platinum drug resistance (Table 1).

**Table 1:** Basic characteristics of patients in HGSOC tissues and the relationship between ALYREF Expression and Clinicopathological Characteristics.

Characteristics	Total	Expression of ALYREF		χ ²	*P*-value
		High (24)	Low (37)		
Age					
<50	8	2 (25%)	6 (75%)	0.794	0.37289
≥50	53	22 (41.5%)	31 (58.5%)		
FIGO staging					
I-II	7	4 (57.1%)	3 (42.9%)	1.05	0.30551
III-IV	54	20 (37%)	34 (63%)		
CA125					
<30.2	2	1 (50%)	1 (50%)	0.098	0.75424
≥30.2	59	23 (39%)	36 (61%)		
HE4					
<140	13	6 (46.2%)	7 (53.8%)	0.321	0.57101
≥140	48	18 (37.5%)	30 (62.5%)		
Lymph node metastasis					
Positive	32	14 (43.7%)	18 (56.3%)	0.547	0.45955
Negative	29	10 (34.5%)	19 (65.5%)		
Greater omentum metastasis					
Positive	45	17 (37.8%)	28 (62.2%)	0.176	0.67483
Negative	16	7 (43.8%)	9 (56.2%)		
Ascites					
Positive	52	20 (38.5%)	32 (61.5%)	0.115	0.73452
Negative	9	4 (44.4%)	5 (55.6%)		
BRCA1/2 mutation					
Positive	17	5 (29.4%)	12 (70.6%)	0.974	0.32368
Negative	44	19 (43.2%)	25 (56.8%)		
Platinum resistance					
Positive	8	6 (75%)	2 (25%)	4.905	**0.02678**
Negative	53	18 (34%)	35 (66%)		

Immunohistochemical score ≥6 is considered high expression. The bolded row in the table is to emphasize significant results.

### Single-cell sequencing identifies a subpopulation of platinum-resistant ovarian cancer cells characterized by elevated ALYREF expression and activation of the WNT signaling pathway

**Fig. 2:**
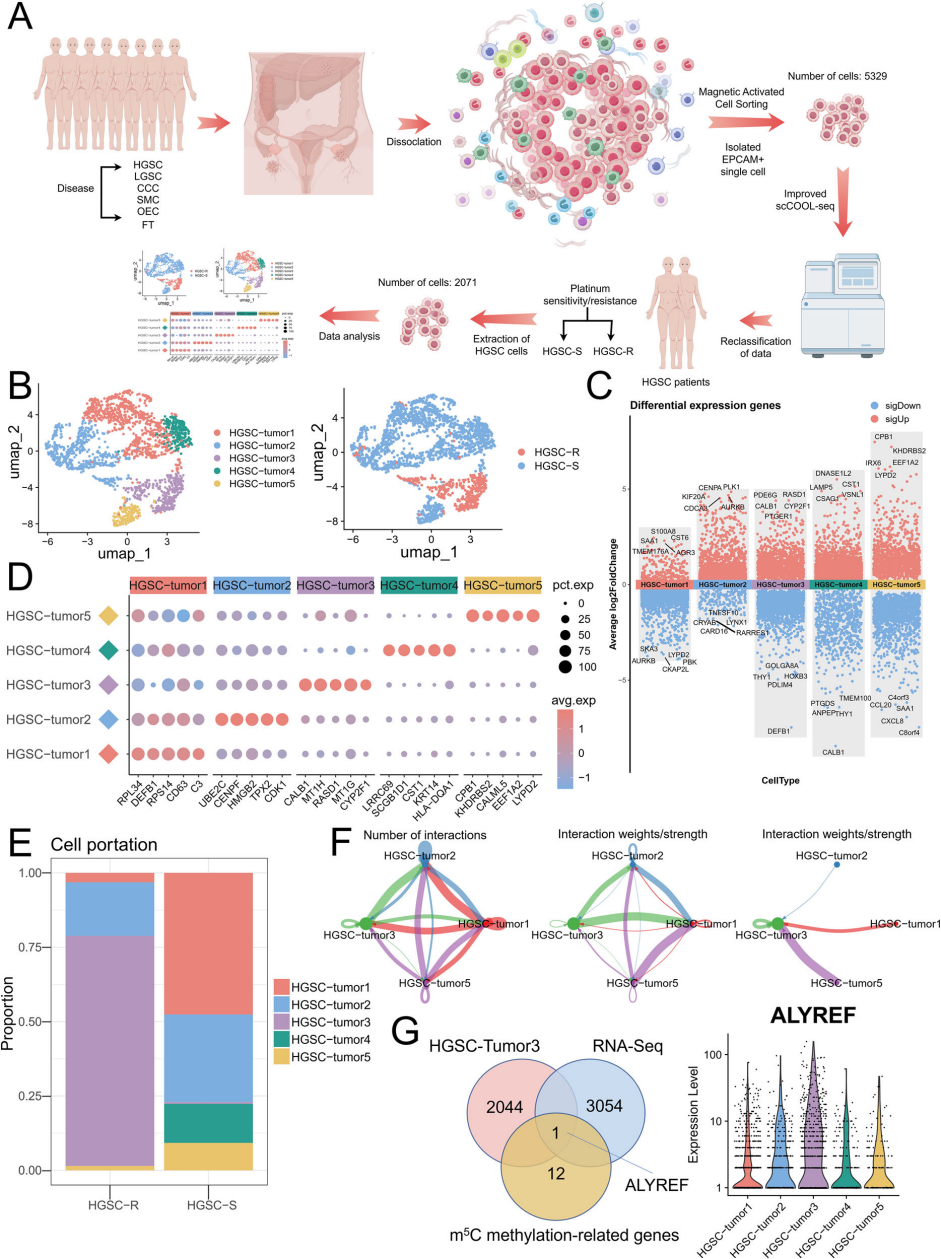
Single-cell sequencing identifies a platinum-resistant ovarian cancer subpopulation with elevated ALYREF expression and Wnt/β-Catenin pathway activation. **A** Simple workflow diagram illustrating the single-cell sequencing analysis by FigDraw. **B** Uniform Manifold Approximation and Projection (UMAP) plot presenting single-cell sequencing data. **C** Bar graph showing maker genes in 5 clusters. **D** The 5 most significant genes across 5 clusters. **E** Cellular distribution of the 5 cell clusters within the patient sample. **F** Network diagram illustrating the interconnections between HGSC-tumor3 and other subtypes. **G** Venn diagram illustrating overlapping DEGs identified among HGSC-tumor3, RNA-Seq in A2780/DDP and parental cells, and m^5^C methylation-related genes.

Detailed single-cell sequencing methods have been previously described in our study [4]. We reanalyzed the data, extracting only cells from HGSC types and further categorizing them into cisplatin-sensitive HGSC (HGSC-S) and cisplatin-resistant HGSC (HGSC-R) based on patient response to cisplatin (Figs. 2A and S2A, B). HGSC-R cells accounted for 19.85% of the total cell population (Fig. S2C). Subsequent clustering analysis divided the single cells into 5 distinct clusters (Figs. 2B–D and S2D, E), among which the HGSC-tumor3 cluster contained the highest proportion of HGSC-R cells (Fig. 2E). We then analyzed the DEGs of HGSC-tumor3 and found that metallothioneins were significantly elevated (MT1H, MT1G, MT1E, MT1F, MT1X, MT2A) (Fig. S3A, B). Since metallothioneins are known to mediate cisplatin resistance by inactivating the drug and promoting DNA repair through glutathione interactions [34], these findings confirm that HGSC-tumor3 is a major cisplatin-resistant subpopulation warranting further investigation. We next used the CellChat software package to analyze intercellular communication within the HGSC-R group. HGSC-tumor3 exhibited extensive interactions with both HGSC-tumor1 and HGSC-tumor2 (Figs. 2F and S4A), with the WNT signaling pathway showing significant activity between HGSC-tumor3 and both HGSC-tumor1 and HGSC-tumor2 (Fig. S4B, E). Additional signaling between HGSC-tumor3 and other HGSC cell populations was primarily mediated by the MK and PTN pathways, which are closely linked to tumor progression (Fig. S4C–E). The results indicate that HGSC-tumor3 might have a pivotal part in the mechanisms of platinum resistance in ovarian cancer. Further analysis identified DEGs shared between cisplatin-resistant A2780/DDP cells and HGSC-tumor3, revealing consistent upregulation of the m⁵C methylation-binding protein ALYREF. This convergence reinforces the hypothesis that ALYREF is crucial in the development of cisplatin resistance in ovarian cancer (Fig. 2G).

### ALYREF may be involved in oncogenic function and cisplatin resistance formation in ovarian cancer

**Fig. 3:**
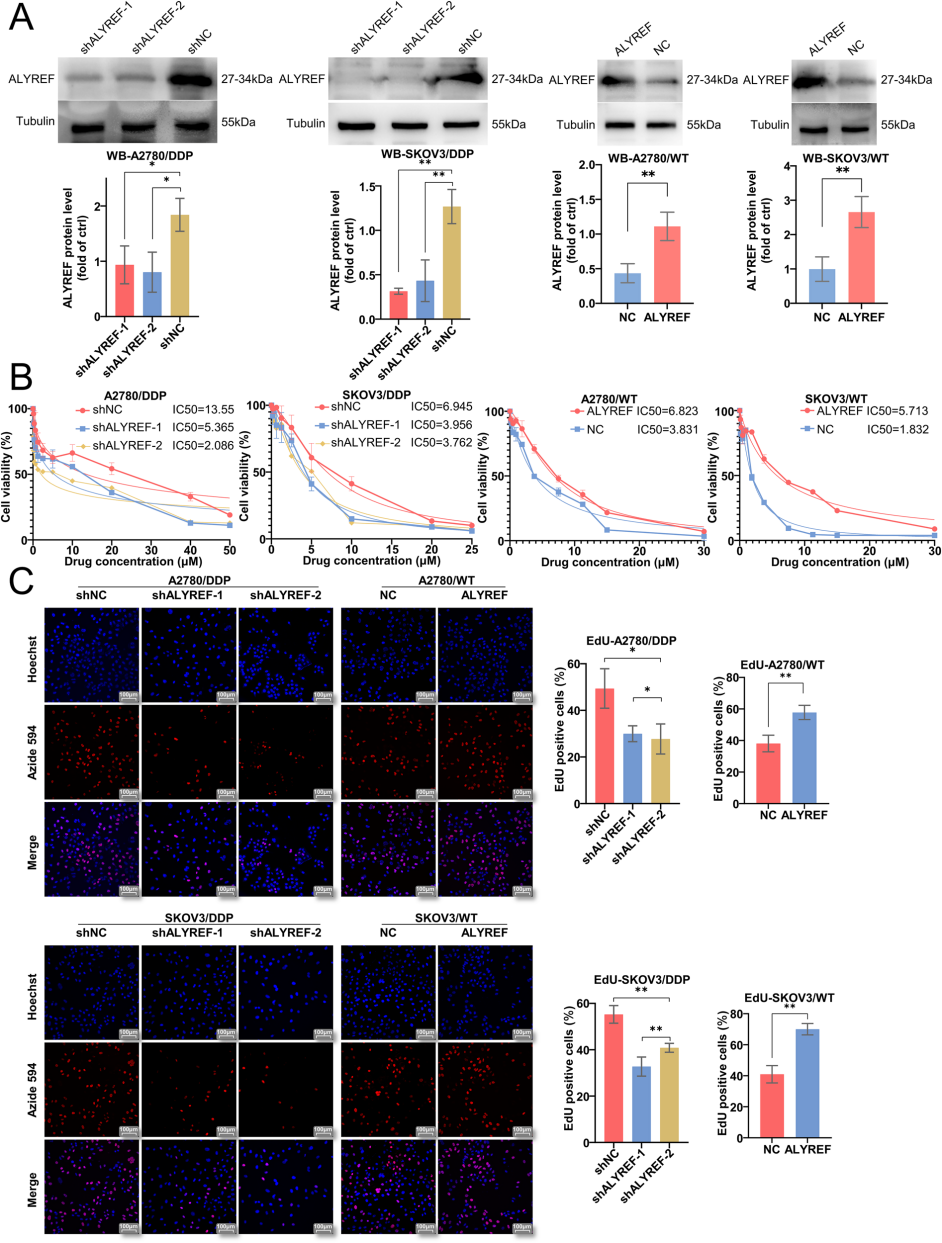
ALYREF may contribute to oncogenic processes and the development of cisplatin resistance in ovarian cancer. **A** Western blot assay of ALYREF knockdown and overexpression in ovarian cancer cells. **B** CCK-8 assay measuring cisplatin resistance in ALYREF knockdown and overexpression ovarian cancer cells. **C** The EdU assay was performed to examine the association between ALYREF expression and cellular proliferative activity. Data are displayed as mean ± SD; *: *p* < 0.05. **: *p* < 0.01.

To elucidate the functional significance of ALYREF in cisplatin-resistant ovarian cancer, we systematically examined its role in malignant phenotypes. Stable knockdown cell lines were generated in A2780/DDP and SKOV3/DDP cells, whereas overexpression cell lines were established in A2780/WT and SKOV3/WT cells. (Figs. 3A and S1B). ALYREF knockdown significantly increased cisplatin sensitivity in both A2780/DDP and SKOV3/DDP cells, whereas overexpression conferred resistance (Figs. 3B and S1C). CCK-8 assays demonstrated that ALYREF depletion attenuated proliferation in both cell lines, whereas overexpression promoted proliferative capacity (Fig. S1D). Consistent with these findings, colony formation capacity was impaired upon ALYREF knockdown but enhanced by overexpression (Fig. S1E). EdU incorporation assays corroborated these results, showing diminished proliferation following ALYREF knockdown and augmented proliferation upon overexpression (Fig. 3C). Transwell assays revealed that ALYREF knockdown suppressed both migratory and invasive capacities, whereas overexpression potentiated these malignant behaviors (Fig. S5A, B).

### ALYREF promotes cisplatin resistance in ovarian cancer by activating the Wnt/β-catenin signaling pathway

**Fig. 4:**
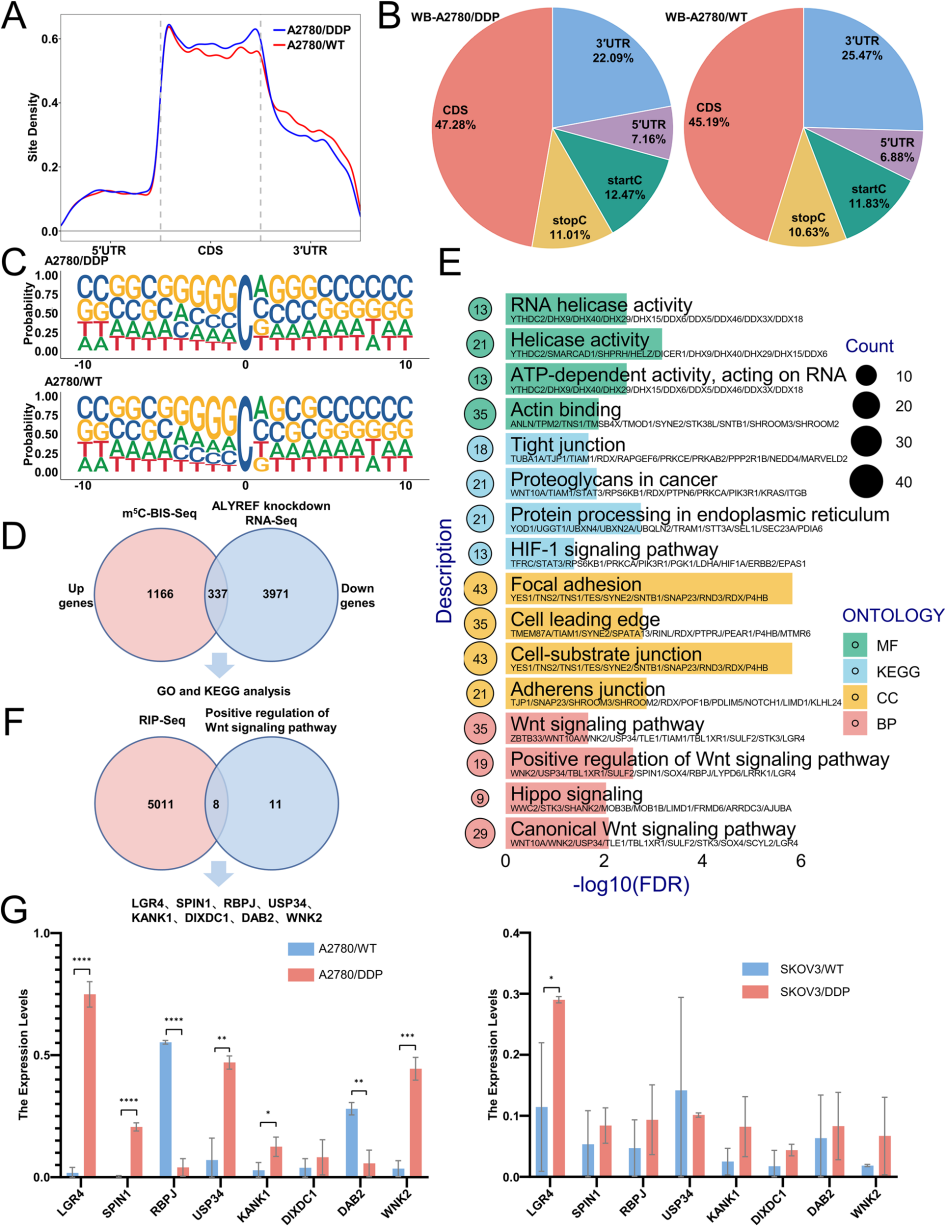
ALYREF promotes cisplatin resistance in ovarian cancer by activating the Wnt/β-catenin signaling pathway. **A** Peak distribution of m^5^C modifications in A2780/DDP and parental cells. **B** Overall differential distribution of m^5^C modifications, illustrated as a pie chart, in A2780/DDP and parental cells. **C** Sequence context analysis of the m^5^C sites, displaying the nucleotide probabilities for 10 bases upstream and downstream. **D** RNA-Seq analysis of ALYREF knockdown A2780/DDP cells and schematic representation of overlapping genes identified by m^5^C-BIS-Seq in A2780/DDP and parental cells. **E** KEGG and GO enrichment analysis of genes identified in both RNA-Seq and m^5^C-BIS-Seq datasets. **F** Intersection of RIP-Seq data with genes positively regulating the Wnt signaling pathway, depicted schematically. **G** qPCR detection of LGR4, SPIN1, RBPJ, USP34, KANK1, DIXDC1, DAB2, and WNK2 expression in A2780/DDP and SKOV3/DDP ovarian cancer cells. Data are displayed as mean ± SD; *: *p* < 0.05. **: *p* < 0.01. ***: *p* < 0.001. ****: *p* < 0.0001.

To elucidate the mechanisms underlying ALYREF-mediated cisplatin resistance in ovarian cancer, we conducted RNA-Seq in shALYREF and shNC A2780/DDP cells, along with m^5^C-BIS-Seq in A2780/DDP and parental cells. m^5^C-BIS-Seq revealed that m^5^C methylation was predominantly enriched in CDS and 3’ untranslated regions (Fig. 4A, B). Further analysis showed that m^5^C methylation sites were predominantly located in CG-rich regions (Fig. 4C), consistent with prior findings. We then integrated RNA-Seq data (identifying ALYREF-dependent downregulated genes) with m^5^C-BIS-Seq data (genes with upregulated m^5^C methylation in cisplatin-resistant cells), yielding an overlapping gene set (Fig. 4D). KEGG and GO enrichment analyses of the intersected genes indicated that ALYREF predominantly modulates expression of Wnt signaling pathway genes (Fig. 4E and Table 2). We further validated the regulatory effect of ALYREF on the Wnt/β-Catenin signaling pathway. Western blot analysis of Wnt/β-catenin pathway components revealed that ALYREF knockdown significantly reduced protein levels of β-catenin, c-MYC, and cyclin D1 (Fig. S6A). Nuclear-cytoplasmic fractionation demonstrated nuclear β-catenin downregulation upon ALYREF depletion (Fig. S6B). Immunofluorescence analysis confirmed that ALYREF knockdown reduced both total and nuclear β-catenin levels (Fig. S6C). To identify direct ALYREF targets, we performed RIP-Seq and overlapped the results with ALYREF-dependent Wnt pathway genes. This analysis identified key ALYREF-bound transcripts, including LGR4, SPIN1, RBPJ, USP34, KANK1, DIXDC1, DAB2, and WNK2 (Fig. 4F and Table 3). Validation in cisplatin-resistant ovarian cancer cells revealed that only LGR4 was significantly upregulated (Fig. 4G). We therefore focused on the functional interplay between ALYREF and LGR4.

**Table 2:** KEGG analysis of the differential genes showed that activation of the Wnt signaling pathway was significantly associated with integrated genes of RNA-Seq data (identifying ALYREF-dependent downregulated genes) with m^5^C-BIS-Seq data (genes with upregulated m^5^C methylation in cisplatin-resistant cells).

Pathway	GeneRatio	Count	*p*-value
Regulation of small GTPase-mediated signal transduction	30/719	30	0.002366401
**Positive regulation of Wnt signaling pathway**	**19/719**	**19**	**0.002798259**
Cell–cell junction organization	23/719	23	0.005623191
Protein targeting to vacuole	10/719	10	0.007258535
Positive regulation of canonical NF-kappaB signal transduction	22/719	22	0.008795347
Canonical Wnt signaling pathway	29/719	29	0.008795347
Viral transcription	10/719	10	0.008795347
Positive regulation of canonical Wnt signaling pathway	15/719	15	0.008795347
ERAD pathway	15/719	15	0.009496536
Cell–cell junction maintenance	6/719	6	0.009496536
Epithelial cell development	22/719	22	0.009496536
Protein folding in endoplasmic reticulum	5/719	5	0.009633983
Hippo signaling	9/719	9	0.009929826
Regulation of canonical Wnt signaling pathway	25/719	25	0.009929826
Response to endoplasmic reticulum stress	25/719	25	0.009929826
Regulation of hippo signaling	7/719	7	0.009929826
Regulation of Wnt signaling pathway	29/719	29	0.012431714
Regulation of viral process	18/719	18	0.017278529
Small GTPase-mediated signal transduction	37/719	37	0.020805534
Wnt signaling pathway	35/719	35	0.022851716
Regulation of small GTPase-mediated signal transduction	30/719	30	0.002366401
Positive regulation of Wnt signaling pathway	19/719	19	0.002798259
Cell-cell junction organization	23/719	23	0.005623191
Protein targeting to vacuole	10/719	10	0.007258535

The bolded row in the table is to emphasize the direction of the study.

**Table 3:** RNA expression and m^5^C modification of WNT signaling pathway-related genes in cisplatin-resistant ovarian cancer cells.

Gene name	RNA-Seq		m^5^C-BIS-Seq			
	log_2_(FC)	*p*-value	Chromosome number	Methylation sites	Avg-methRate	Gene-region
**LGR4**	**1.1786**	**3.73E-07**	**chr11**	**27369140**	**0.2**	**CDS**
				**27368164**	**0.2**	**CDS**
				**27472140**	**0.167**	**startC**
				**27367941**	**0.15**	**stopC**
SPIN1	1.29808	5.27E-07	chr9	88476547	0.125	3UTR
				88462692	0.133	CDS
KANK1	1.35649	3.44E-06	chr9	711871	0.2	CDS
				712909	0.167	CDS
WNK2	1.87568	4.14E-06	chr9	93292573	0.154	CDS
				93292592	0.133	CDS
RBPJ	1.59935	1.26E-05	chr4	26362585	0.167	startC
				26430762	0.143	CDS
DIXDC1	1.37305	0.001026	chr11	112020110	0.143	3UTR
				112019021	0.105	stopC
USP34	1.02081	0.001254	chr2	61288862	0.182	CDS
				61288854	0.167	CDS
DAB2	1.74207	0.002994	chr5	39388338	0.184	CDS
				39377255	0.175	CDS

The bolded row in the table is to emphasize the direction of the study.

### NSUN2 mediates m^5^C methylation in the CDS of LGR4 mRNA to promote cisplatin resistance in ovarian cancer

**Fig. 5:**
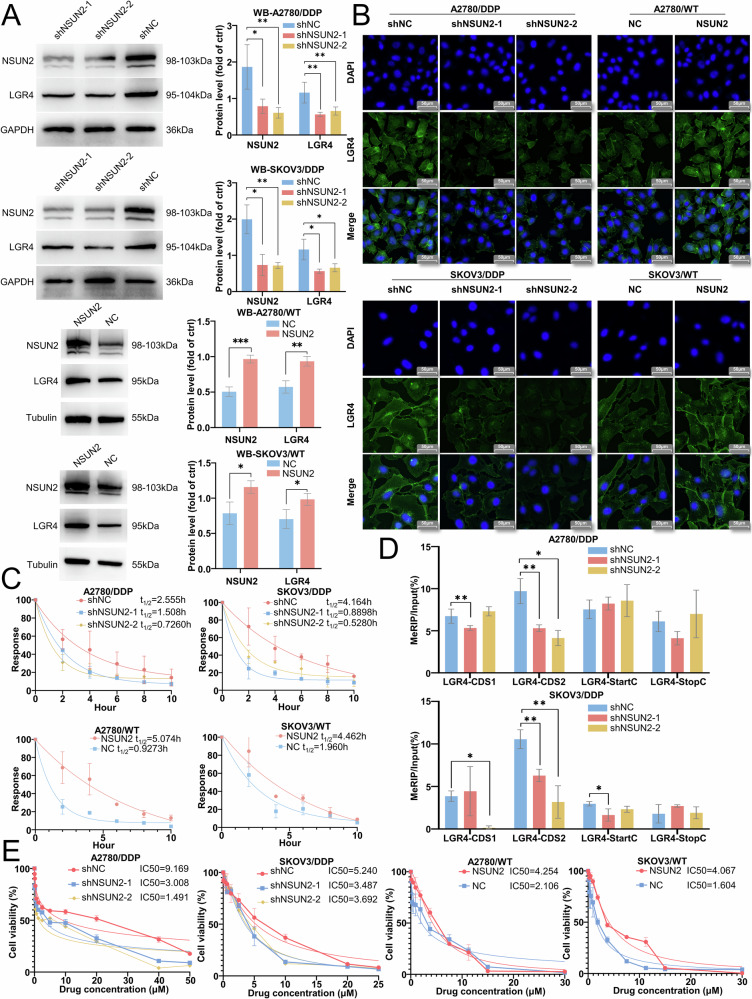
NSUN2 mediates m^5^C methylation in the CDS region of LGR4 mRNA to promote cisplatin resistance in ovarian cancer. **A** Western blot analysis of LGR4 protein expression in A2780/DDP and SKOV3/DDP cells with NSUN2 knockdown, and in A2780/WT and SKOV3/WT cells with NSUN2 overexpression. **B** Immunofluorescence assay to assess the impact of NSUN2 knockdown and overexpression on LGR4 protein levels in ovarian cancer cells. **C** Actinomycin D assay assessing the impact of NSUN2 knockdown and overexpression on LGR4 mRNA stability. **D** m^5^C-RIP and qRT-PCR assays detecting the influence of NSUN2 on the m^5^C methylation level of LGR4 mRNA. **E** CCK-8 assay measuring cisplatin resistance in NSUN2 knockdown and overexpression ovarian cancer cells. Data are displayed as mean ± SD; *: *p* < 0.05. **: *p* < 0.01.

As a methylation reader protein, ALYREF does not directly catalyze RNA m^5^C modification. According to previous reports, ALYREF functions in coordination with the methyltransferase NSUN2, which installs m^5^C modifications that are subsequently recognized by ALYREF. To determine the role of NSUN2 in ALYREF-dependent LGR4 regulation, we generated NSUN2-knockdown and -overexpressing cell lines. NSUN2 knockdown markedly decreased LGR4 mRNA and protein levels, whereas overexpression enhanced them (Figs. 5A,B and S7A). Moreover, NSUN2 knockdown reduced LGR4 mRNA stability and global RNA m^5^C methylation levels, whereas overexpression resulted in their elevation (Figs. 5C and S7B). To pinpoint the specific region of LGR4 mRNA affected by NSUN2-mediated methylation, four primer pairs were designed targeting m^5^C sites in the CDS, start codon (StartC), and stop codon (StopC) regions, based on m^5^C-BIS-Seq data. m^5^C-RIP-qPCR showed selective reduction of m^5^C methylation in the LGR4 CDS region upon NSUN2 knockdown, with minimal changes in StartC or StopC regions (Fig. 5D). Furthermore, NSUN2 knockdown sensitized A2780/DDP and SKOV3/DDP cells to cisplatin, whereas overexpression enhanced resistance in A2780/WT and SKOV3/WT cells (Figs. 5E and S7C). Collectively, these data demonstrate that NSUN2 establishes CDS-specific m^5^C marks on LGR4 mRNA to enable ALYREF recognition, thereby promoting cisplatin resistance in ovarian cancer.

### ALYREF recognizes m^5^C modifications on LGR4 mRNA through its RNA-binding domain, increasing transcript stability and subsequent activity of Wnt/β-catenin signaling in ovarian cancer cells

**Fig. 6:**
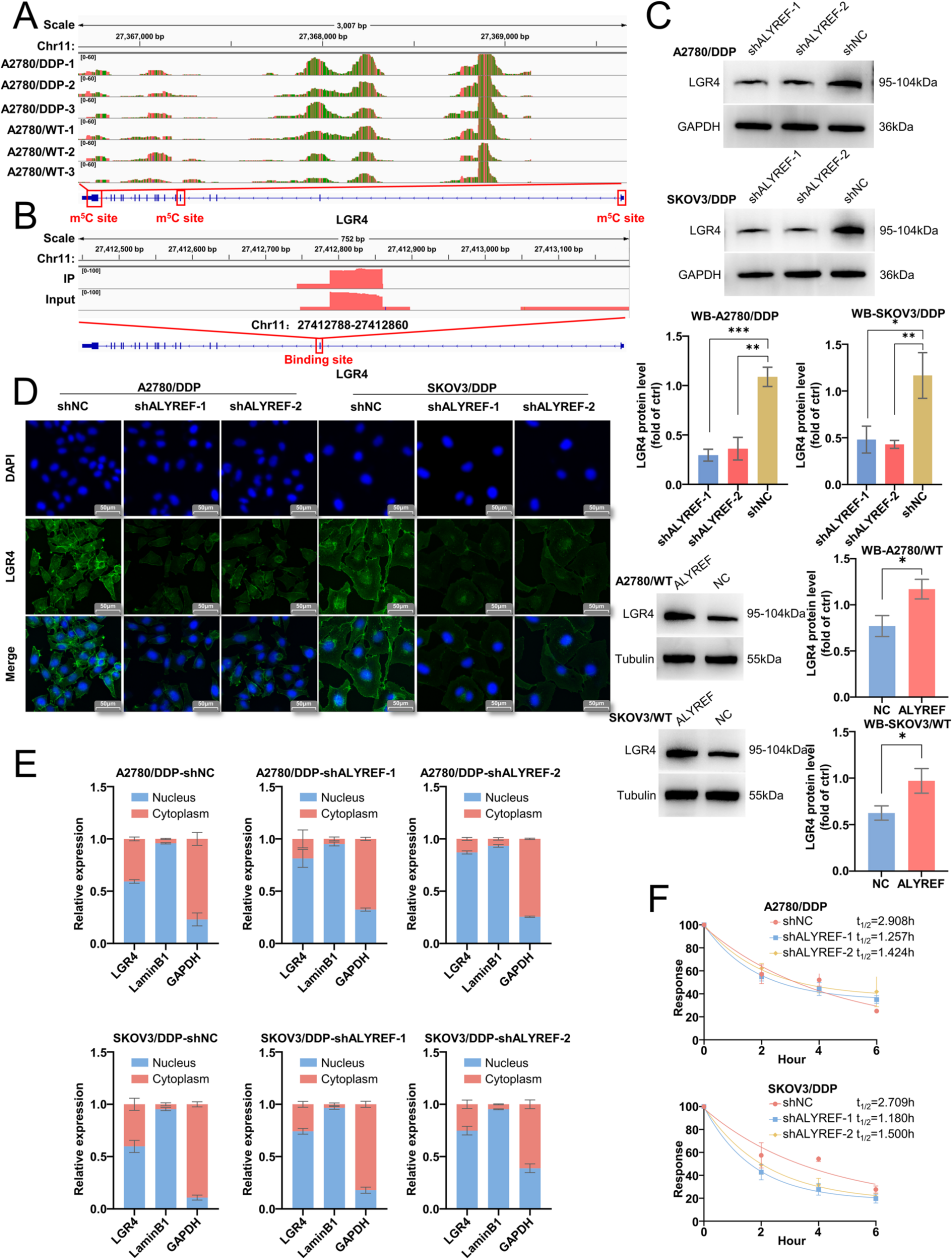
ALYREF promotes the Wnt/β-Catenin pathway by enhancing LGR4 mRNA stability. **A** IGV visualization of m^5^C enrichment peak changes in the CDS, StopC and StartC regions of LGR4 mRNA in A2780/DDP and parental cells. **B** IGV visualization of LGR4 enrichment peaks in the Input group and ALYREF-IP group. **C** Western blot analysis of LGR4 protein expression in ovarian cancer cells with ALYREF knockdown and overexpression. **D** Immunofluorescence assay to assess the impact of ALYREF knockdown on LGR4 protein levels in A2780/DDP and SKOV3/DDP cells. **E** Subcellular distribution of LGR4 RNA in the cytoplasm and nucleus. **F** Actinomycin D assay assessing the impact of ALYREF knockdown on LGR4 mRNA stability. Data are displayed as mean ± SD; *: *p* < 0.05. **: *p* < 0.01. ***: *p* < 0.001.

Based on m^5^C-BIS-Seq analysis, we identified elevated m^5^C methylation at multiple sites within the CDS, StartC, and StopC regions of LGR4 mRNA in A2780/DDP cells (Fig. 6A). RIP-Seq of ALYREF further confirmed a direct interaction between LGR4 mRNA and the methylation-binding protein ALYREF (Fig. 6B). To investigate the functional relationship between ALYREF and LGR4, we knocked down or overexpressed ALYREF in ovarian cancer cells. ALYREF knockdown led to significant downregulation of LGR4 mRNA and protein expression, while its overexpression resulted in increased expression (Figs. 6C, D and S7D, E). ALYREF functions not only as an m^5^C reader but also as a key mRNA export factor, playing an essential role in the nuclear–cytoplasmic transport of mRNA. To investigate this function, we separately extracted cytoplasmic and nuclear RNA and observed that cytoplasmic LGR4 RNA levels were reduced following ALYREF knockdown (Fig. 6E). Furthermore, ALYREF knockdown markedly reduced the stability of LGR4 mRNA (Figs. 6F and S8A), suggesting that ALYREF may stabilize LGR4 transcripts and promote nuclear export via recognition of m^5^C-modified sites. To confirm whether LGR4 functions downstream of ALYREF, we generated A2780/DDP and SKOV3/DDP cell lines with ALYREF knockdown and concurrent LGR4 overexpression. The results showed that partial upregulation of LGR4 partially restored the reduced cisplatin resistance induced by ALYREF knockdown (Fig. S8B, C). Moreover, functional assays demonstrated that upregulation of LGR4 also rescued the inhibitory effects of ALYREF knockdown on cell proliferation, migration, and invasion (Fig. S8D, E). In addition, overexpression of LGR4 restored activity of the Wnt/β-Catenin pathway suppressed by ALYREF knockdown (Fig. S8F).

**Fig. 7:**
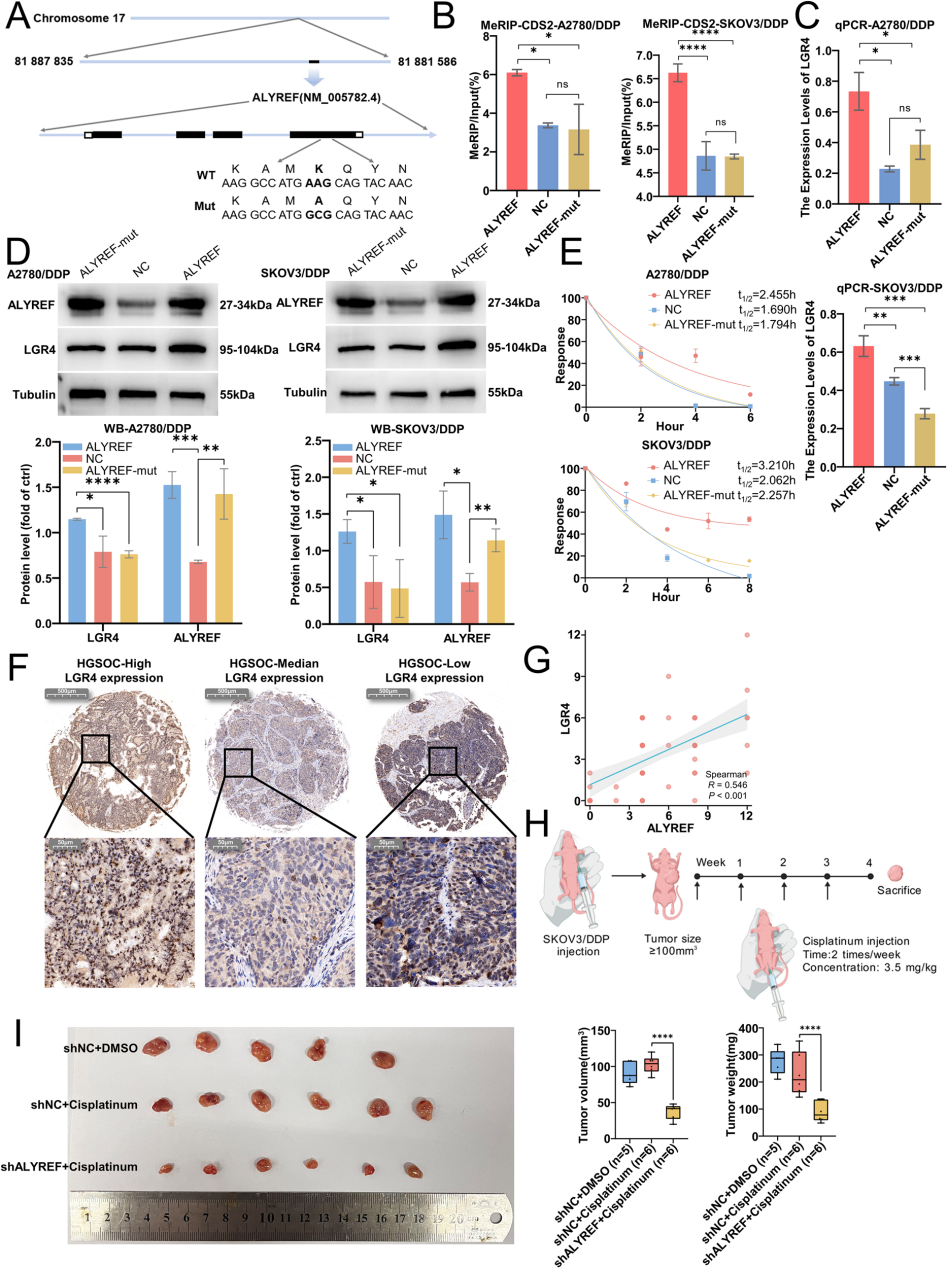
ALYREF confers cisplatin resistance in ovarian cancer by specifically binding m^5^C-modified LGR4 mRNA. **A** Schematic diagram of the ALYREF m^5^C methylation binding site mutation. **B** m^5^C-RIP and qRT-PCR assays detecting the influence of ALYREF on the m^5^C methylation level of LGR4 mRNA. **C** qPCR detection of LGR4 RNA expression in the influence of ALYREF. **D** Western blot analysis of LGR4 protein expression in ALYREF-overexpressing A2780/DDP and SKOV3/DDP cells, and in cells with mutated ALYREF binding sites. **E** Actinomycin D assay assessing the impact of ALYREF on LGR4 mRNA stability. **F** Representative images of IHC staining of LGR4 expression levels in tissue microarrays. **G** Correlation analysis between ALYREF and LGR4 protein expression levels. **H** Schematic representation of the subcutaneous tumor model in cisplatin-resistant ovarian cancer-bearing mice by Generic Diagramming Platform (GDP) [41]. **I** Final diameter and weight of the subcutaneous tumors. Data are displayed as mean ± SD; *: *p* < 0.05. **: *p* < 0.01. ***: *p* < 0.001. ****: *p* < 0.0001.

**Fig. 8:**
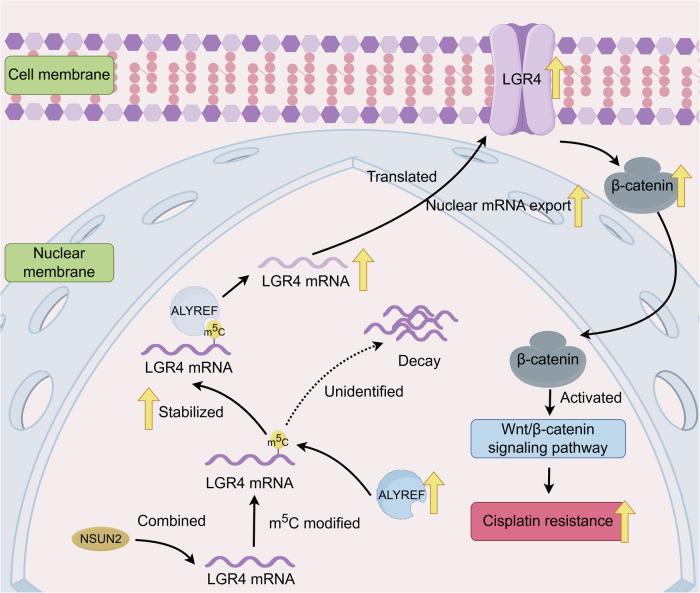
NSUN2/ALYREF/LGR4 signaling axis promotes cisplatin resistance in ovarian cancer by activating the Wnt/β-catenin pathway through m⁵C-dependent stabilization of LGR4 mRNA. **A** This schematic illustrates that NSUN2-mediated m⁵C modification of LGR4 mRNA is recognized and bound by ALYREF, which enhances the stability and nuclear export of LGR4 transcripts. **B** Increased LGR4 protein expression subsequently activates β-catenin signaling, resulting in β-catenin accumulation and downstream activation of the Wnt/β-catenin pathway. **C** Activation of this signaling cascade ultimately leads to enhanced cisplatin resistance in ovarian cancer. Solid arrows represent activation events, dotted arrows indicate potential intermediate processes, and yellow arrows denote upregulated molecular changes.

We next mutated the m^5^C recognition site on ALYREF (Fig. 7A). While ALYREF overexpression enhanced m^5^C methylation in the CDS region of LGR4 mRNA, this effect was abolished in cells expressing the mutated version (Fig. 7B). Correspondingly, ALYREF overexpression failed to upregulate LGR4 expression or mRNA stability in the mutant group (Fig. 7C–E), confirming the functional importance of m^5^C site recognition by ALYREF. Analysis of the cBioPortal database revealed a positive correlation between ALYREF and LGR4 expression in HGSC samples (Fig. S8G). Immunohistochemical analysis of HGSC tissue microarrays (Fig. S9A) showed a moderate correlation between ALYREF and LGR4 protein expression (Fig. 7F, G). Finally, in a cisplatin-resistant subcutaneous tumor mouse model (Fig. 7H), ALYREF knockdown significantly reduced cisplatin resistance and led to downregulation of ALYREF, LGR4, and β-Catenin protein levels in tumor tissues (Figs. 7I and S9B, C). These findings indicate that ALYREF promotes cisplatin resistance in ovarian cancer by recognizing m^5^C-modified sites on LGR4 mRNA, thereby enhancing its stability, facilitating nuclear export, and activating the Wnt/β-catenin pathway (Fig. 8).

## Discussion

In our study, we identified a potential role for ALYREF in platinum resistance through reanalysis of RNA-Seq data from cisplatin-resistant ovarian cancer cell lines and single-cell sequencing data from patient samples. Subsequent investigations revealed that ALYREF promotes platinum resistance in ovarian cancer cells by activating the Wnt/β-Catenin pathway via the NSUN2/ALYREF/LGR4 signaling axis. LGR4 is a key regulator of the Wnt/β-catenin pathway, which is crucial for the development of platinum resistance, primarily through its interaction with the RSPO family of proteins that amplify Wnt signaling. For instance, Wang et al. showed that the Wnt/β-catenin pathway affects the chemotherapeutic sensitivity of gastric cancer by regulating the iron death process [35]. Chi and Song et al. demonstrated that the Wnt/β-catenin pathway is associated with cisplatin sensitivity in cervical and ovarian cancer [36, 37]. Although ALYREF has not been specifically reported regarding chemotherapeutic drug resistance, Wang et al. found that NSUN2 modulated gefitinib resistance in EGFR-mutated non-small cell lung cancers, and Chen et al. demonstrated that NSUN2 functions as a glucose sensor to maintain tumorigenicity and confer immunotherapeutic resistance [38, 39]. This suggests that m^5^C methylation modifications positively influence tumor chemotherapy and immunotherapy outcomes.

In our study, we observed a puzzling phenomenon: binding site mutation and overexpression of ALYREF alter the level of m^5^C modification in the CDS region of LGR4 mRNA in platinum-resistant ovarian cancer cells. However, as a reader rather than a writer, ALYREF theoretically should not directly influence m^5^C addition. To investigate this discrepancy, we reviewed the literature and proposed the following possible explanations. First, ALYREF, as an mRNA m^5^C-binding protein, primarily facilitates the nuclear export of m^5^C-modified RNA. Reduced ALYREF expression may impair its ability to bind and stabilize m^5^C-modified RNAs, leading to their degradation and a subsequent decrease in intracellular m^5^C levels. Second, ALYREF may regulate the expression or activity of m^5^C methyltransferases through an indirect mechanism. Previous studies have shown that both ALYREF and NSUN2 are upregulated in urothelial carcinoma of the bladder, suggesting a possible synergistic effect [10]. Although the precise mechanism remains unclear, this finding implies that ALYREF might influence RNA m^5^C methylation by modulating the expression or stability of NSUN2. These observations support the idea that m^5^C methylation is a dynamic process involving writers, readers, and erasers. As a reader, ALYREF may participate in a feedback loop where its binding to m^5^C-modified RNAs could enhance the activity of methyltransferases. Conversely, reduced ALYREF expression may disrupt this regulatory balance, ultimately leading to decreased m^5^C levels. Future studies should further elucidate these mechanisms to better understand ALYREF’s role in RNA m^5^C methylation.

Additionally, our study found that ALYREF-mediated stabilization of LGR4 mRNA depends on its recognition of the m^5^C modification site. This mechanism resembles that of YBX1, which regulates E2F1 mRNA stability through phase separation [40]. However, whether ALYREF enhances its function via phase separation or through complex formation with other proteins remains unclear. Given that m^5^C methylation has been shown to establish positive feedback loops, it is worth investigating whether ALYREF is involved in a similar regulatory network.

In summary, this study provides the first evidence that ALYREF recognizes m^5^C modifications on LGR4 mRNA, thereby enhancing its stability and promoting nuclear export, which in turn activates the Wnt signaling pathway. This mechanism ultimately contributes to platinum resistance in ovarian cancer. These findings not only expand our understanding of RNA epigenetic modifications in tumor drug resistance but also provide new insights for targeted ovarian cancer therapy. Future research will focus on determining whether targeting ALYREF can reverse platinum resistance and exploring the development of small-molecule inhibitors of ALYREF, with the goal of providing novel therapeutic strategies for drug-resistant ovarian cancer patients.

## Supplementary information

Supplementary figures and table

Supplementary digital image of Western blotting

## Data Availability

Raw single-cell sequencing data from our previous studies have been deposited in the Genome Sequence Archive (GSA) under accession number HRA000360, and they are available upon reasonable request. The processed single-cell sequencing data have been submitted to the GEO database (GSE189955). RNA-Seq, BIS-Seq and RIP-Seq sequencing raw data in this study have been uploaded to Sequence Read Archive (SRA) with the login number: PRJNA1265627. For legal reasons and to protect patient privacy, data pertaining to patients’ clinical information is not publicly accessible.

## References

[1] Konstantinopoulos PA, Matulonis UA. Clinical and translational advances in ovarian cancer therapy. Nat Cancer. 2023;4:1239–57. 37653142 10.1038/s43018-023-00617-9

[2] Baert T, Ferrero A, Sehouli J, O’Donnell DM, González-Martín A, Joly F, et al. The systemic treatment of recurrent ovarian cancer revisited. Ann Oncol. 2021;32:710–25. 33675937 10.1016/j.annonc.2021.02.015

[3] El-Agwany A. Chemotherapy response assessment using ultrasound in ovarian cancer. Gynecology Obstet Clin Med. 2024;4:e000026.

[4] Wang Y, Xie H, Chang X, Hu W, Li M, Li Y, et al. Single-cell dissection of the multiomic landscape of high-grade serous ovarian cancer. Cancer Res. 2022;82:3903–16. 35969151 10.1158/0008-5472.CAN-21-3819 PMC9627134

[5] Nombela P, Miguel-López B, Blanco S. The role of m(6)A, m(5)C and Ψ RNA modifications in cancer: novel therapeutic opportunities. Mol Cancer. 2021;20:18. 33461542 10.1186/s12943-020-01263-w PMC7812662

[6] Chellamuthu A, Gray SG. The RNA methyltransferase NSUN2 and its potential roles in cancer. Cells. 2020;9:1758. 32708015 10.3390/cells9081758 PMC7463552

[7] Balachander K, Priyadharsini JV, Roy A, Paramasivam A. Emerging role of RNA m^5^c modification in cardiovascular diseases. J Cardiovasc Transl Res. 2023;16:598–605. 36318418 10.1007/s12265-022-10336-8

[8] Blaze J, Navickas A, Phillips HL, Heissel S, Plaza-Jennings A, Miglani S, et al. Neuronal Nsun2 deficiency produces tRNA epitranscriptomic alterations and proteomic shifts impacting synaptic signaling and behavior. Nat Commun. 2021;12:4913. 34389722 10.1038/s41467-021-24969-x PMC8363735

[9] Xu J, Liu X, Chen Y, Wang Y, Liu T, Yi P. RNA 5-methylcytosine regulators contribute to metabolism heterogeneity and predict prognosis in ovarian cancer. Front Cell Dev Biol. 2022;10:807786. 35372362 10.3389/fcell.2022.807786 PMC8971725

[10] Wang N, Chen RX, Deng MH, Wei WS, Zhou ZH, Ning K, et al. m(5)C-dependent cross-regulation between nuclear reader ALYREF and writer NSUN2 promotes urothelial bladder cancer malignancy through facilitating RABL6/TK1 mRNAs splicing and stabilization. Cell Death Dis. 2023;14:139. 36806253 10.1038/s41419-023-05661-y PMC9938871

[11] Nulali J, Zhang K, Long M, Wan Y, Liu Y, Zhang Q, et al. ALYREF-mediated RNA 5-methylcytosine modification promotes hepatocellular carcinoma progression via stabilizing EGFR mRNA and pSTAT3 activation. Int J Biol Sci. 2024;20:331–46. 38164181 10.7150/ijbs.82316 PMC10750289

[12] Jin Y, Yao J, Fu J, Huang Q, Luo Y, You Y, et al. ALYREF promotes the metastasis of nasopharyngeal carcinoma by increasing the stability of NOTCH1 mRNA. Cell Death Dis. 2024;15:578. 39117671 10.1038/s41419-024-06959-1 PMC11310353

[13] Shi M, Zhang H, Wu X, He Z, Wang L, Yin S, et al. ALYREF mainly binds to the 5’ and the 3’ regions of the mRNA in vivo. Nucleic Acids Res. 2017;45:9640–53. 28934468 10.1093/nar/gkx597 PMC5766156

[14] Zhao Y, Xing C, Peng H. ALYREF (Aly/REF export factor): a potential biomarker for predicting cancer occurrence and therapeutic efficacy. Life Sci. 2024;338:122372. 38135116 10.1016/j.lfs.2023.122372

[15] Zheng P, Li N, Zhan X. Ovarian cancer subtypes based on the regulatory genes of RNA modifications: novel prediction model of prognosis. Front Endocrinol. 2022;13:972341. 10.3389/fendo.2022.972341 PMC9760687 36545327

[16] McMellen A, Woodruff ER, Corr BR, Bitler BG, Moroney MR. Wnt signaling in gynecologic malignancies. Int J Mol Sci.2020;21:4272. 32560059 10.3390/ijms21124272 PMC7348953

[17] Nagaraj AB, Joseph P, Kovalenko O, Singh S, Armstrong A, Redline R, et al. Critical role of Wnt/β-catenin signaling in driving epithelial ovarian cancer platinum resistance. Oncotarget. 2015;6:23720–34. 26125441 10.18632/oncotarget.4690 PMC4695147

[18] Iluta S, Nistor M, Buruiana S, Dima D. Wnt signaling pathway in tumor biology. Genes.2024;15:1597. 39766864 10.3390/genes15121597 PMC11675244

[19] Perumalsamy YKN, Warrier NK, Perumalsamy S, Dharmarajan LR. A. Wnt antagonist as therapeutic targets in ovarian cancer. Int J Biochem Cell Biol. 2022;145:106191. 35272015 10.1016/j.biocel.2022.106191 PMC7616886

[20] Targeting LGR4-Wnt activates ferroptosis and reverses drug resistance in colorectal cancer. Nat Cancer. 2024;5:542–3. 10.1038/s43018-023-00714-9 38355778

[21] Chowanadisai W, Messerli SM, Miller DH, Medina JE, Hamilton JW, Messerli MA, et al. Cisplatin-resistant spheroids model clinically relevant survival mechanisms in ovarian tumors. PLoS ONE. 2016;11:e0151089. 26986722 10.1371/journal.pone.0151089 PMC4795743

[22] Meng Y, Chen CW, Yung MMH, Sun W, Sun J, Li Z, et al. DUOXA1-mediated ROS production promotes cisplatin resistance by activating ATR-Chk1 pathway in ovarian cancer. Cancer Lett. 2018;428:104–16. 29704517 10.1016/j.canlet.2018.04.029 PMC7474466

[23] Schneider CA, Rasband WS, Eliceiri KW. NIH Image to ImageJ: 25 years of image analysis. Nat Methods. 2012;9:671–5. 22930834 10.1038/nmeth.2089 PMC5554542

[24] Livak KJ, Schmittgen TD. Analysis of relative gene expression data using real-time quantitative PCR and the 2(-Delta Delta C(T)) Method. Methods. 2001;25:402–8. 11846609 10.1006/meth.2001.1262

[25] Satija R, Farrell JA, Gennert D, Schier AF, Regev A. Spatial reconstruction of single-cell gene expression data. Nat Biotechnol. 2015;33:495–502. 25867923 10.1038/nbt.3192 PMC4430369

[26] Hu C, Li T, Xu Y, Zhang X, Li F, Bai J, et al. CellMarker 2.0: an updated database of manually curated cell markers in human/mouse and web tools based on scRNA-seq data. Nucleic Acids Res. 2023;51:D870–d6. 36300619 10.1093/nar/gkac947 PMC9825416

[27] Jin S, Guerrero-Juarez CF, Zhang L, Chang I, Ramos R, Kuan CH, et al. Inference and analysis of cell-cell communication using CellChat. Nat Commun. 2021;12:1088. 33597522 10.1038/s41467-021-21246-9 PMC7889871

[28] Ritchie ME, Phipson B, Wu D, Hu Y, Law CW, Shi W, et al. Limma powers differential expression analyses for RNA-sequencing and microarray studies. Nucleic Acids Res. 2015;43:e47. 25605792 10.1093/nar/gkv007 PMC4402510

[29] Wu T, Hu E, Xu S, Chen M, Guo P, Dai Z, et al. clusterProfiler 4.0: A universal enrichment tool for interpreting omics data. Innovation. 2021;2:100141. 34557778 10.1016/j.xinn.2021.100141 PMC8454663

[30] wickham H. ggplot2: Elegant Graphics for Data Analysis. New York: Springer-Verlag. 2016.

[31] Maeser D, Gruener RF, Huang RS. oncoPredict: an R package for predicting in vivo or cancer patient drug response and biomarkers from cell line screening data. Brief Bioinform. 2021;22:bbab260. 10.1093/bib/bbab260 PMC8574972 34260682

[32] Geeleher P, Cox NJ, Huang RS. Clinical drug response can be predicted using baseline gene expression levels and in vitro drug sensitivity in cell lines. Genome Biol. 2014;15:R47. 24580837 10.1186/gb-2014-15-3-r47 PMC4054092

[33] Yang S, Zhou D, Zhang C, Xiang J, Xi X. Function of m(5)C RNA methyltransferase NOP2 in high-grade serous ovarian cancer. Cancer Biol Ther. 2023;24:2263921. 37800580 10.1080/15384047.2023.2263921 PMC10561575

[34] Si M, Lang J. The roles of metallothioneins in carcinogenesis. J Hematol Oncol. 2018;11:107. 30139373 10.1186/s13045-018-0645-x PMC6108115

[35] Wang Y, Zheng L, Shang W, Yang Z, Li T, Liu F, et al. Wnt/beta-catenin signaling confers ferroptosis resistance by targeting GPX4 in gastric cancer. Cell Death Differ. 2022;29:2190–202. 35534546 10.1038/s41418-022-01008-w PMC9613693

[36] Chi C, Hou W, Zhang Y, Chen J, Shen Z, Chen Y, et al. PDHB-AS suppresses cervical cancer progression and cisplatin resistance via inhibition on Wnt/β-catenin pathway. Cell Death Dis. 2023;14:90. 36750722 10.1038/s41419-022-05547-5 PMC9905568

[37] Song Z, Liao C, Yao L, Xu X, Shen X, Tian S, et al. miR-219-5p attenuates cisplatin resistance of ovarian cancer by inactivating Wnt/β-catenin signaling and autophagy via targeting HMGA2. Cancer Gene Ther. 2023;30:596–607. 36494581 10.1038/s41417-022-00574-y

[38] Wang Y, Wei J, Feng L, Li O, Huang L, Zhou S, et al. Aberrant m5C hypermethylation mediates intrinsic resistance to gefitinib through NSUN2/YBX1/QSOX1 axis in EGFR-mutant non-small-cell lung cancer. Mol Cancer. 2023;22:81. 37161388 10.1186/s12943-023-01780-4 PMC10169458

[39] Chen T, Xu ZG, Luo J, Manne RK, Wang Z, Hsu CC, et al. NSUN2 is a glucose sensor suppressing cGAS/STING to maintain tumorigenesis and immunotherapy resistance. Cell Metab. 2023;35:1782–98.e8. 37586363 10.1016/j.cmet.2023.07.009 PMC10726430

[40] Liu X, Wei Q, Yang C, Zhao H, Xu J, Mobet Y, et al. RNA m(5)C modification upregulates E2F1 expression in a manner dependent on YBX1 phase separation and promotes tumor progression in ovarian cancer. Exp Mol Med. 2024;56:600–15. 38424195 10.1038/s12276-024-01184-4 PMC10984993

[41] Jiang S, Li H, Zhang L, Mu W, Zhang Y, Chen T, et al. Generic Diagramming Platform (GDP): a comprehensive database of high-quality biomedical graphics. Nucleic Acids Res. 2025;53:D1670–d6. 39470721 10.1093/nar/gkae973 PMC11701665

