# Supplementary material for: Multi-omics analysis reveals that ALYREF-mediated m5C modification promotes platinum resistance in ovarian cancer via the NSUN2/ALYREF/LGR4 axis
Source: Cell Death Dis. 2025 Dec 5;17(1):77. doi: 10.1038/s41419-025-08310-8 (PMC12827963; doi:10.1038/s41419-025-08310-8)
Supplement: Supplementary file 1 — Supplementary figures and table [file 41419_2025_8310_MOESM1_ESM.pdf]

Supplementary figures and table

Table S1 Primers' sequences used in qPCR were as follows:

Gene Symbol	Primers' sequences
GAPDH	Forward primer: 5’-CAGGGCTGCTTTTAACTCTGGTAA-3’<br>Reverse primer: 5’-GGGTGGAATCATATTGGAACATGT-3’
ALYREF	Forward primer: 5’-TCTCAGACGCCGATATTCAGG-3’<br>Reverse primer: 5’-GTCTGCTGTTCCTAAGCTGCG-3’
LGR4	Forward primer: 5’-ACTCAAAGTTCTAACGCTCCAG-3’<br>Reverse primer: 5’-AAAGCACTCAGCCCTCGAATG-3’
LaminB1	Forward primer: 5’-AAGCATGAAACGCGCTTGG-3’<br>Reverse primer: 5’-AGTTTGGCATGGTAAGTCTGC-3’
SPIN1	Forward primer: 5’-CAGTGTGGGTCCGAGCAAA-3’<br>Reverse primer: 5’-CAGGGCCATTCCCCTCTTT-3’
KANK1	Forward primer: 5’-GTGCCGAGGAGAACATGAAC-3’<br>Reverse primer: 5’-CTCTAAGCTGTACTTCTAGGCGA-3’
WNK2	Forward primer: 5’-CGCTTCCTCAAGTTCGACATC-3’<br>Reverse primer: 5’-TGGACTCCCAGAAGTCGTAGA-3’
RBPJ	Forward primer: 5’-CGGCCTCCACCTAAACGAC-3’<br>Reverse primer: 5’-TCCATCCACTGCCCATAAGAT-3’
DIXDC1	Forward primer: 5’-GTGCAAAGAGCGAGTCCATTA-3’<br>Reverse primer: 5’-GGTCTCCAGATAGGTTCCAGG-3’
USP34	Forward primer: 5’-TGGCTACATATTCCCGCTGTC -3’<br>Reverse primer: 5’-GCTGCACTCTGTCGTAACTCC-3’
DAB2	Forward primer: 5’-GTAGAAACAAGTGCAACCAATGG-3’<br>Reverse primer: 5’-GCCTTTGAACCTTGCTAAGAGA-3’
LGR4-CDS1	Forward primer: 5’-TGCCAATGCAGGACAGCTG-3’<br>Reverse primer: 5’-ACTGGAAGTTACTGAAGCGAC-3’
LGR4-CDS2	Forward primer: 5’-GGATAGATACTTACAGAACTACCAGG-3’<br>Reverse primer: 5’-GAGGACAGTTTTGAAGGACTTG-3’
LGR4-StartC	Forward primer: 5’-TCGTTTACGCTGAGCCCCTCGGG-3’<br>Reverse primer: 5’-GGGCGGCGGGGGACATCGGA-3’
LGR4-StopC	Forward primer: 5’-GGGGGAAACGGTTACACAC-3’<br>Reverse primer: 5’-CTTCTTGCCAAAGACCTGAG-3’

Figure S1

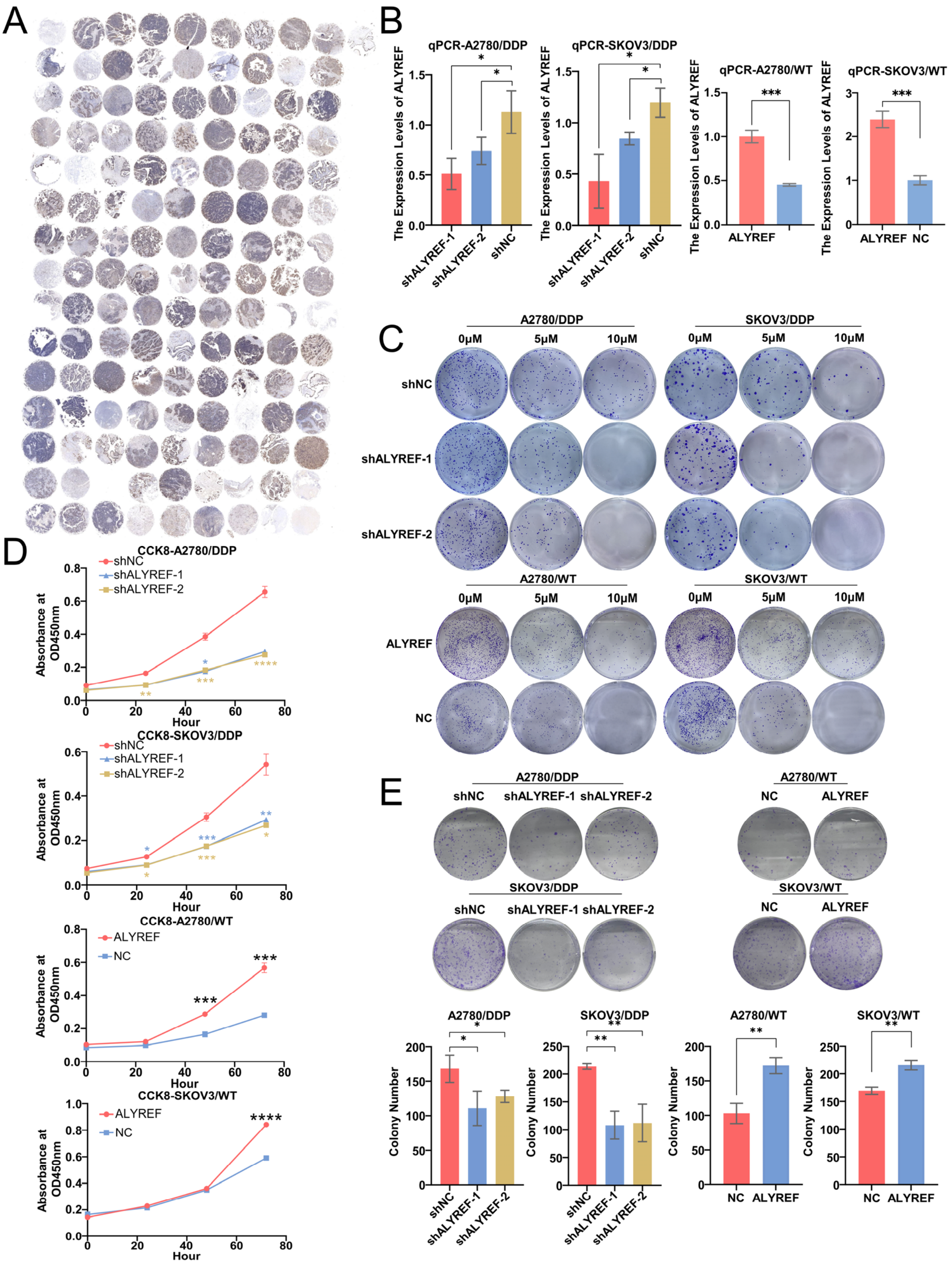

(A) IHC staining revealing ALYREF expression levels in tissue microarrays. (B) qPCR assay of ALYREF knockdown and overexpression in ovarian cancer cells. (C) Clone-forming ability of cisplatin-resistant ovarian cancer cells treated with varying concentrations of cisplatin after ALYREF knockdown and overexpression. (D) The CCK-8 assay was used to assess the relationship between ALYREF expression and cellular growth capacity. (E) The colony formation assay was conducted to evaluate the relationship between ALYREF expression and clonogenic capacity. Data are displayed

as mean ± SD; *: $p < 0.05$. **: $p < 0.01$. ***: $p < 0.001$. ****: $p < 0.0001$.

Figure S2

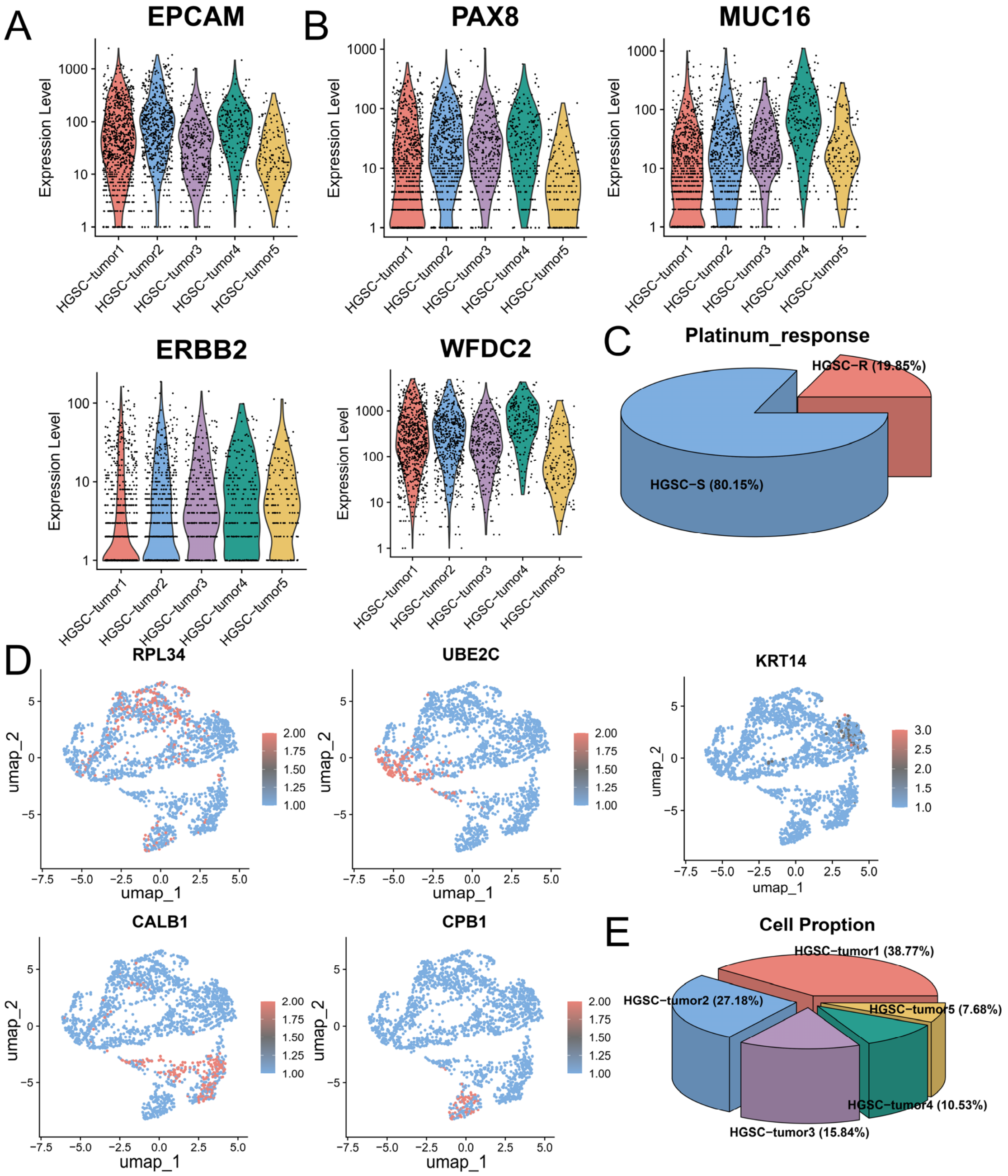

(A) Epithelial cell markers expressed across 5 clusters. (B) Ovarian cancer cell-associated markers were expressed across 5 clusters. (C) Distribution of different groups relative to the total cell count. (D) UMAP distribution of the most significantly different genes among the cell clusters. (E) Distribution of individual cell clusters as a percentage of the total cell population.

Figure S3

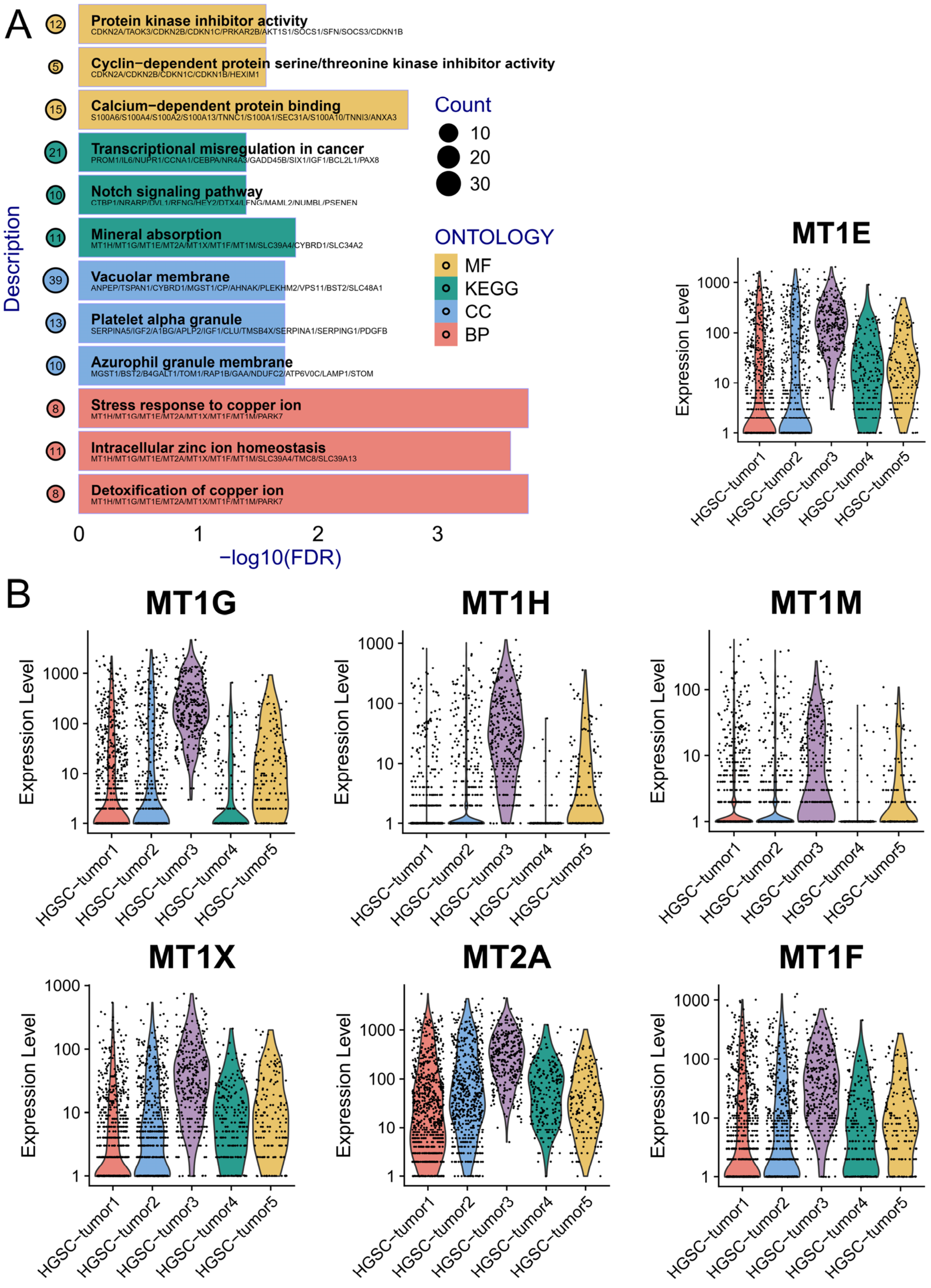

(A) Chordal plots showing the results of KEGG and GO enrichment analysis of HGSC-tumor3. (B) Metallothionein expressed across 5 clusters.

Figure S4

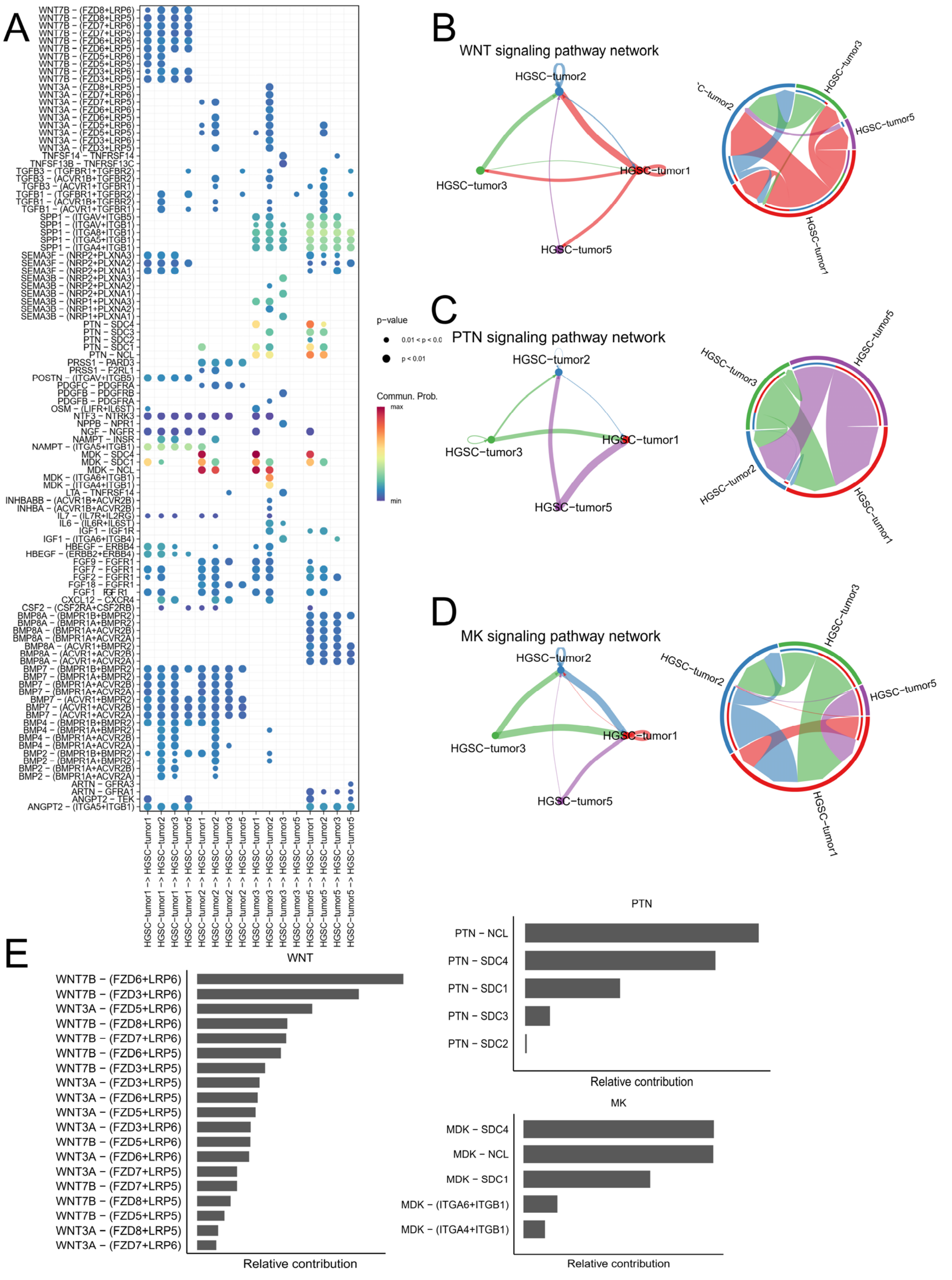

(A) Network diagram depicting cell-cell communication within the HGSC-R group. (B) Network diagram illustrating the WNT pathway connections between HGSC-tumor3 and other subtypes. (C) Network diagram illustrating the PTN pathway connections between HGSC-tumor3 and other subtypes. (D) Network diagram illustrating the MK pathway connections between HGSC-tumor3 and other subtypes. (E) HGSC-tumor3 interacts with ligand receptors associated with the MK, PTN and WNT pathways from other subtypes.

Figure S5

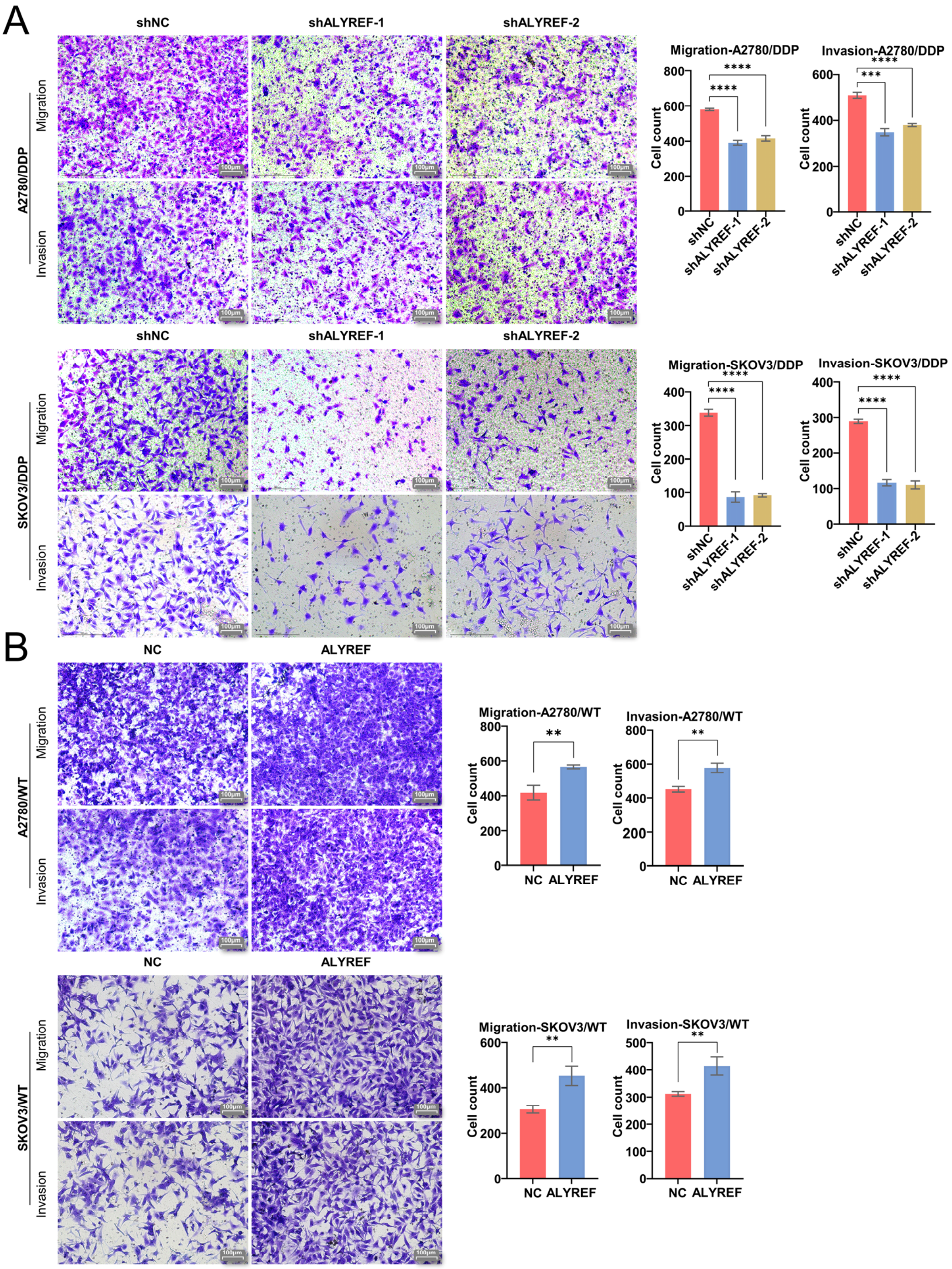

(A-B) Migration and invasion assays were conducted to assess the association between ALYREF expression and the migratory and invasive capabilities of cisplatin-resistant ovarian cancer cells. Data are displayed as mean ± SD; **: $p < 0.01$. ****: $p < 0.0001$.

Figure S6

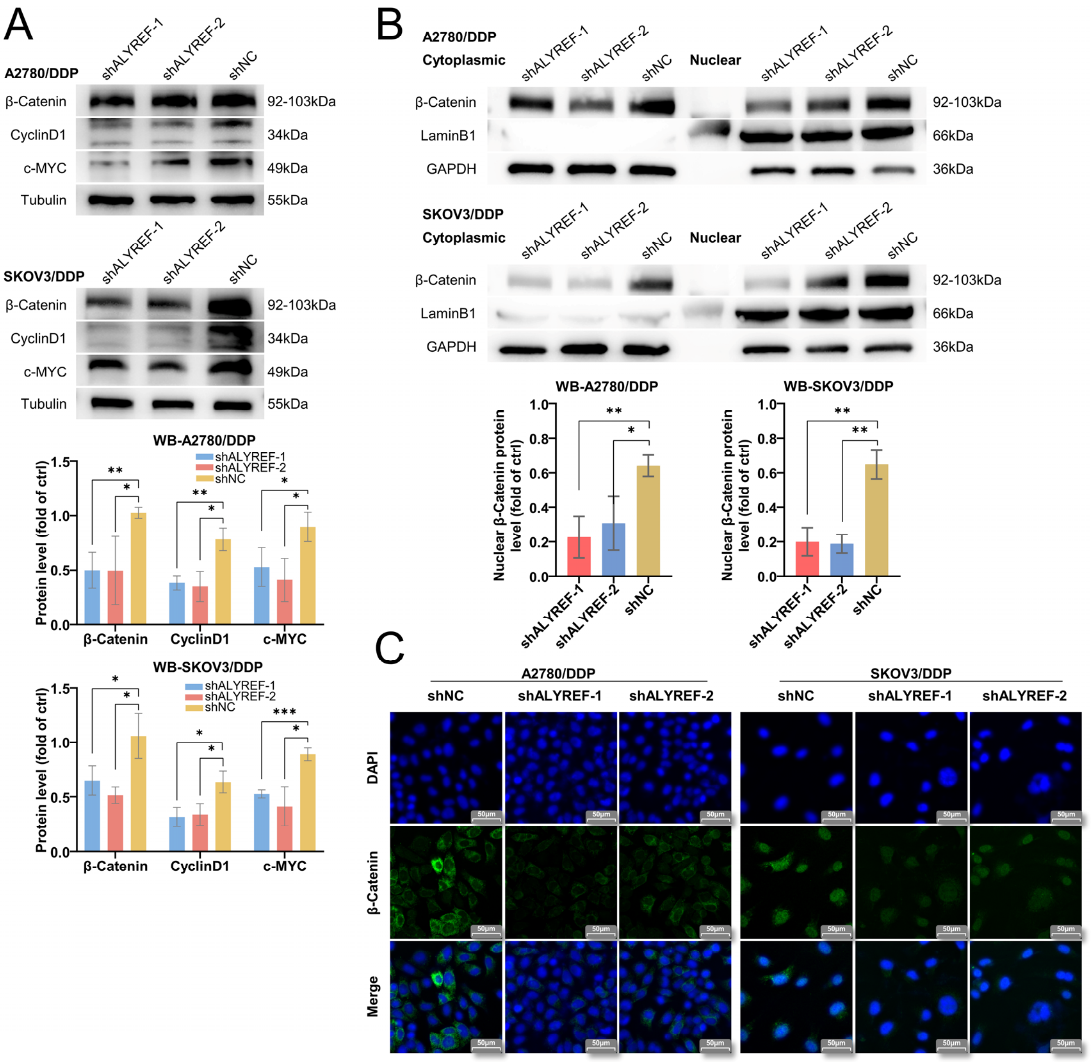

(A) Western blot assay conducted to assess changes in Wnt/β-Catenin pathway-related proteins following ALYREF knockdown. (B) Western blot assay conducted to examine changes in β-Catenin protein expression levels within the nucleus of A2780/DDP and SKOV3/DDP cells following ALYREF knockdown. (C) Immunofluorescence assay to assess the impact of ALYREF knockdown on β-Catenin protein levels in A2780/DDP and SKOV3/DDP cells. Data are displayed as mean ± SD; *: $p < 0.05$. **: $p < 0.01$ ***: $p < 0.001$.

Figure S7

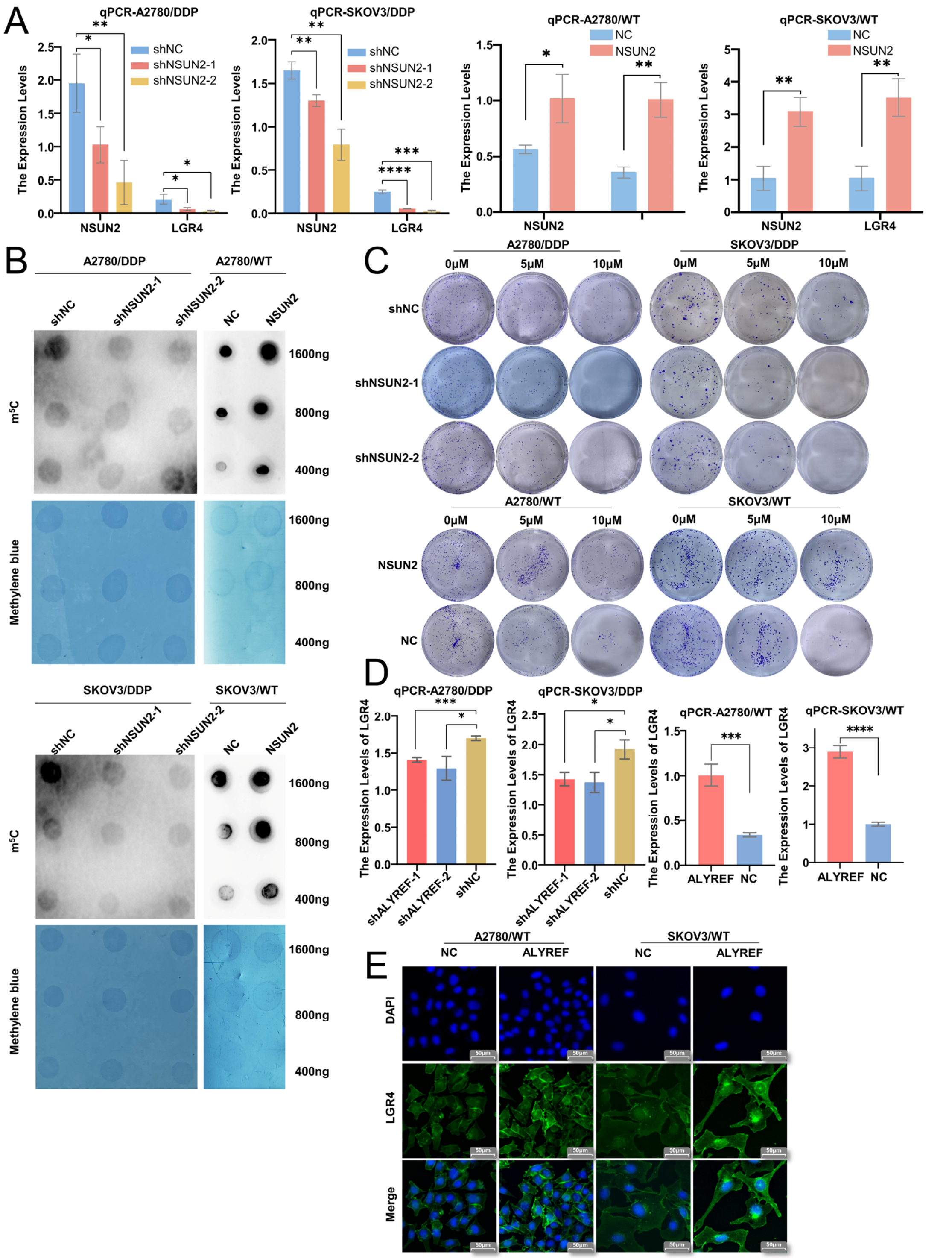

(A) qPCR detection of LGR4 RNA expression in ovarian cancer cells with NSUN2 knockdown and overexpression. (B) Dot blotting assay measuring overall m^5C methylation levels in NSUN2 knockdown and overexpression ovarian cancer cells. (C) Clone-forming ability of cisplatin-resistant ovarian cancer cells treated with varying concentrations of cisplatin after NSUN2 knockdown and overexpression. (D) qPCR detection of LGR4 RNA expression in ovarian cancer cells with ALYREF knockdown and overexpression. (E) Immunofluorescence assay to assess the impact of ALYREF

overexpression on LGR4 protein levels in A2780/WT and SKOV3/WT cells. Data are displayed as mean ± SD; *: $p < 0.05$. **: $p < 0.01$. ***: $p < 0.001$. ****: $p < 0.0001$.

Figure S8

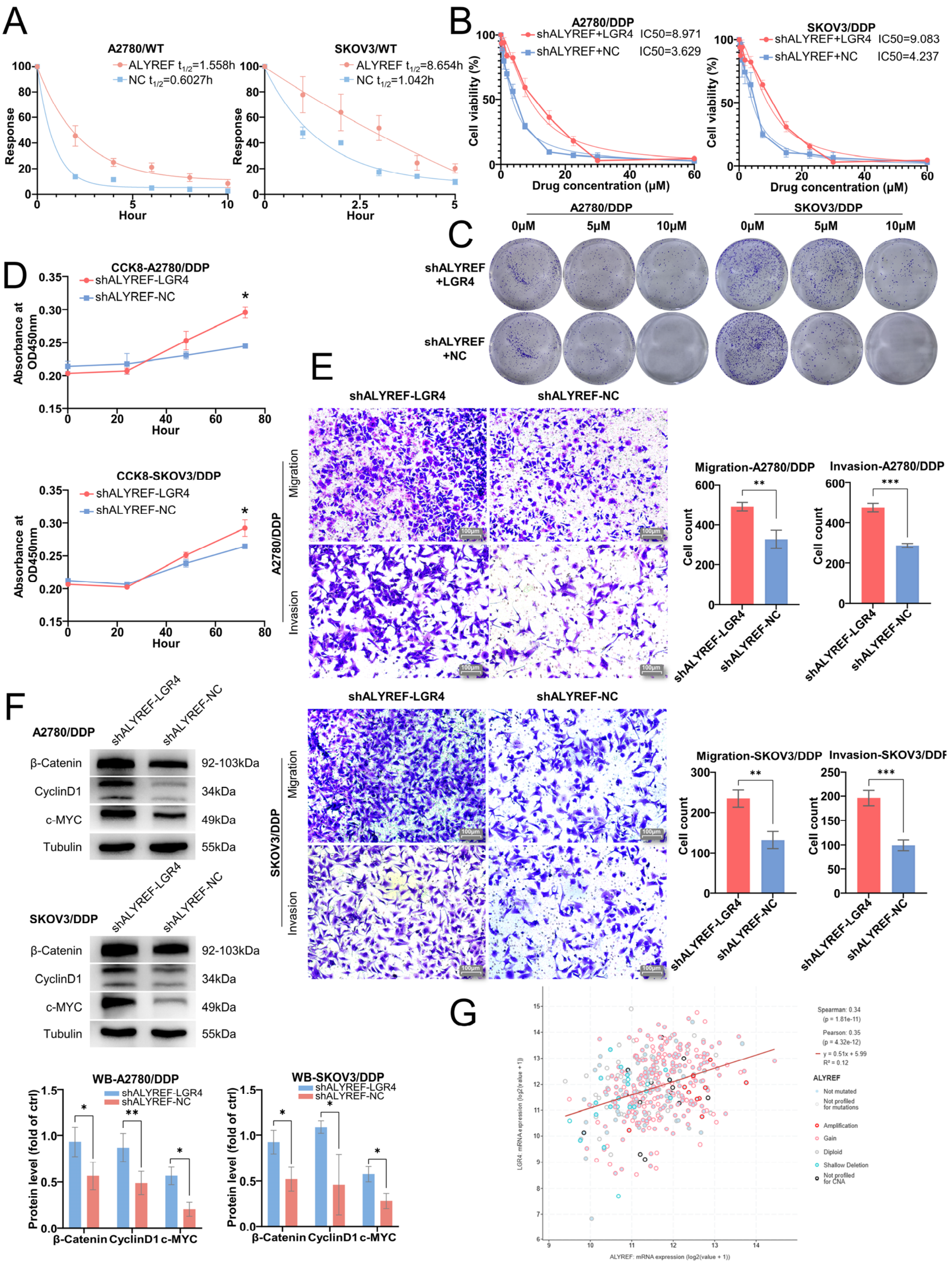

(A) Actinomycin D assay assessing the impact of ALYREF overexpression on LGR4 mRNA stability. (B-C) CCK-8 assay and colony formation assay demonstrates that LGR4 overexpression rescues the reduced cisplatin resistance caused by ALYREF knockdown. (D-E) CCK-8 cell proliferation, Transwell migration, and invasion assays

indicate that overexpression of LGR4 rescues functional deficits caused by ALYREF knockdown. (F) Western blot assay performed to evaluate changes in Wnt/β-Catenin pathway-related proteins after ALYREF knockdown and LGR4 overexpression. (G) Correlation analysis between ALYREF and LGR4 mRNA expression was performed using the cBioPortal online platform. Data are displayed as mean ± SD; *: $p < 0.05$. **: $p < 0.01$.

Figure S9

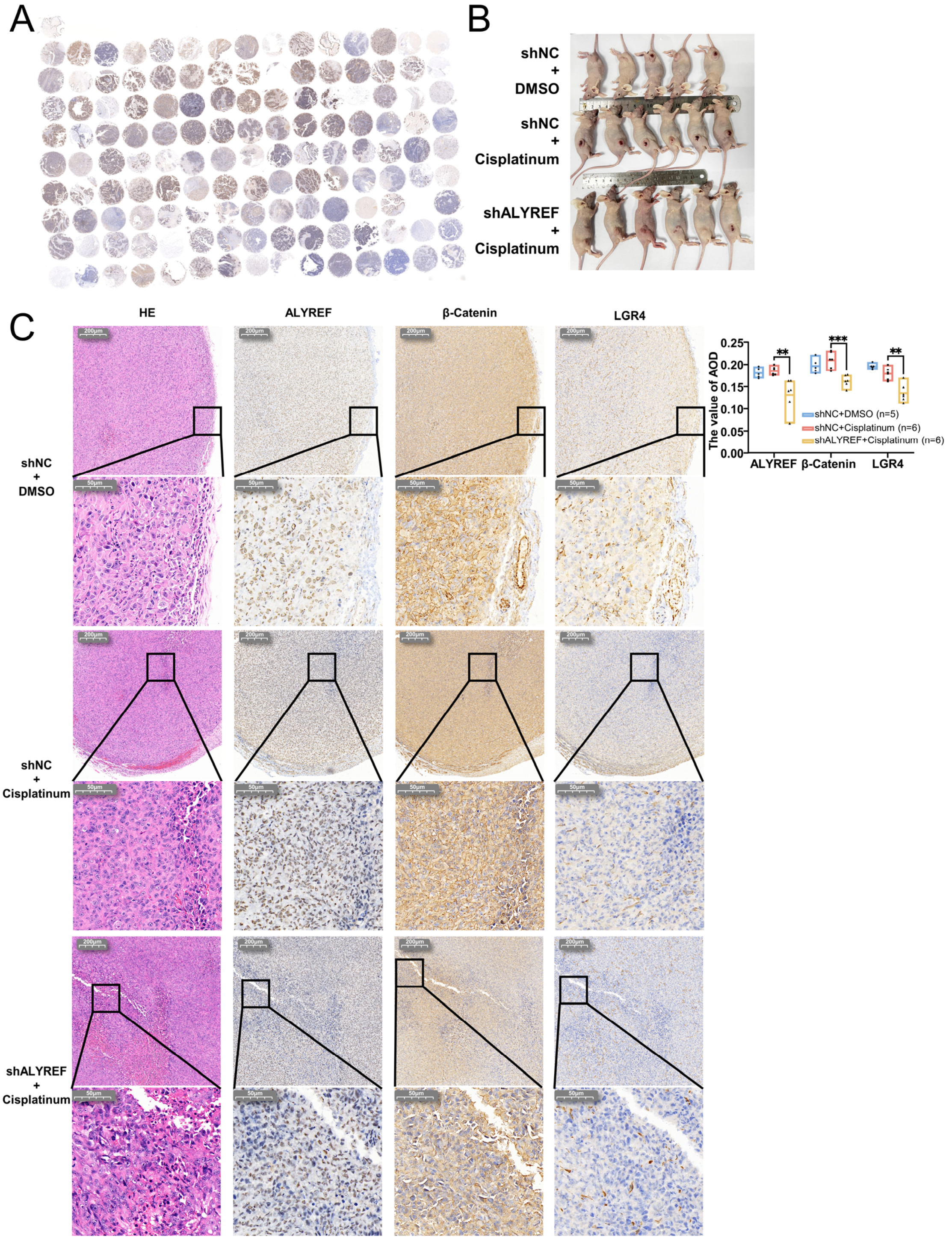

(A) IHC staining revealing LGR4 expression levels in tissue microarrays. (B) Images of mice bearing subcutaneous tumors following cisplatin treatment. (C) Representative figures of IHC staining showing ALYREF, LGR4, and β-Catenin protein levels in the subcutaneous tumors.
