# Supplementary material for: Multi-omics analysis reveals that ALYREF-mediated m5C modification promotes platinum resistance in ovarian cancer via the NSUN2/ALYREF/LGR4 axis
Source: Cell Death Dis. 2025 Dec 5;17(1):77. doi: 10.1038/s41419-025-08310-8 (PMC12827963; doi:10.1038/s41419-025-08310-8)
Supplement: Supplementary file 2 — Supplementary digital image of Western blotting [file 41419_2025_8310_MOESM2_ESM.pdf]

Supplementary original figures of Western blotting

The images shown in the article we have marked with red boxes.
The following images were taken by Imaging Systems (ChemiDox, Bio-Rad, USA):

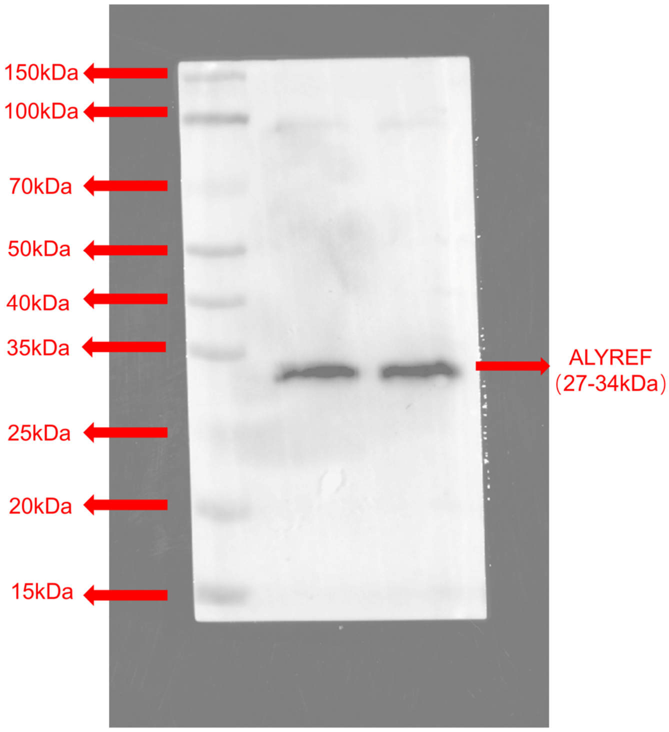

Figure 1 Fuller-length, original, and unprocessed Western blot images of Anti-ALYREF antibody to confirm specific detection of the target antigen. (Predicted band size: 27 kDa, Observed band size: 27-34 kDa)

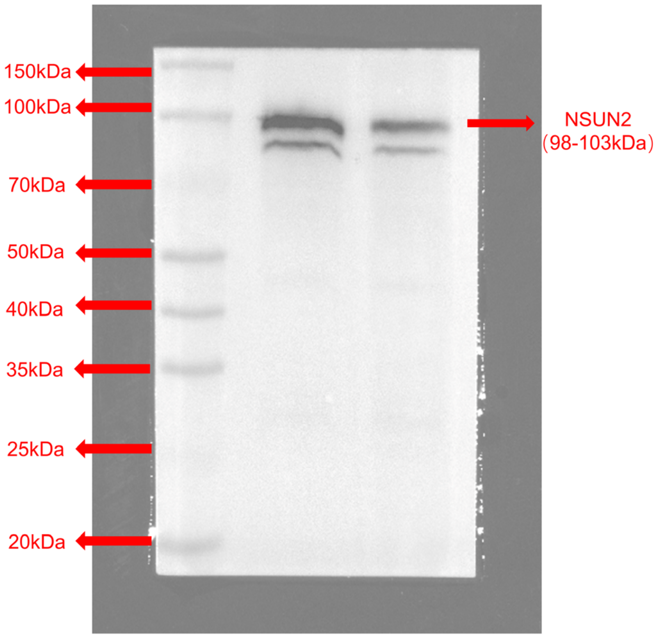

Figure 2 Fuller-length, original, and unprocessed Western blot images of Anti-NSUN2 antibody to confirm specific detection of the target antigen. (Predicted band size: 86 kDa, Observed band size: 98-103 kDa)

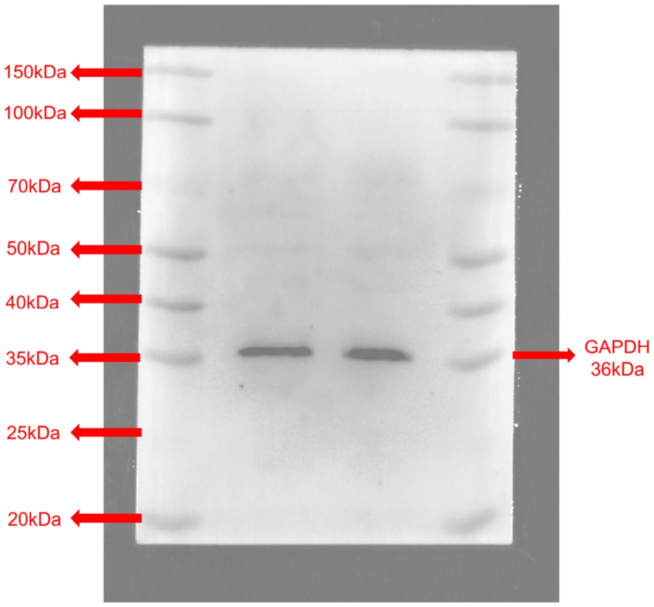

Figure 3 Fuller-length, original, and unprocessed Western blot images of Anti-GAPDH antibody to confirm specific detection of the target antigen. (Predicted band size: 36 kDa, Observed band size: 36 kDa)

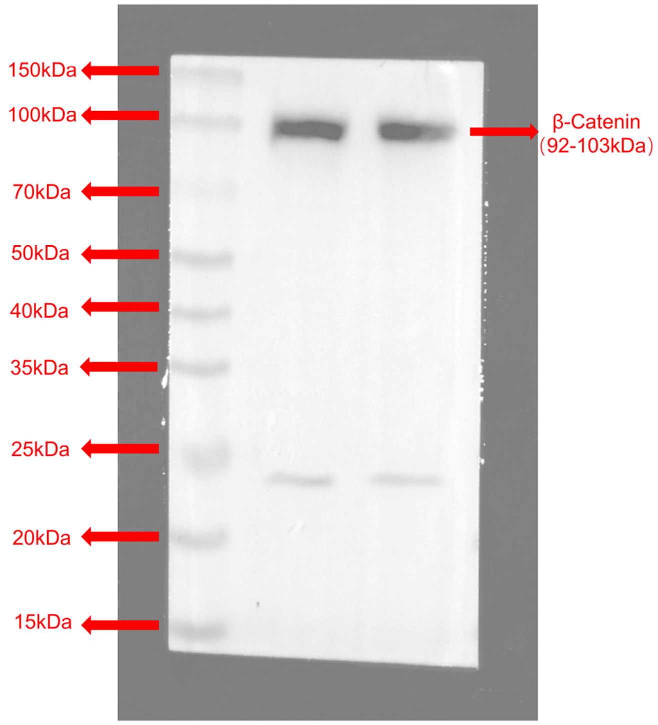

Figure 4 Fuller-length, original, and unprocessed Western blot images of Anti-β-Catenin antibody to confirm specific detection of the target antigen. (Predicted band size: 92 kDa, Observed band size: 92-103 kDa)

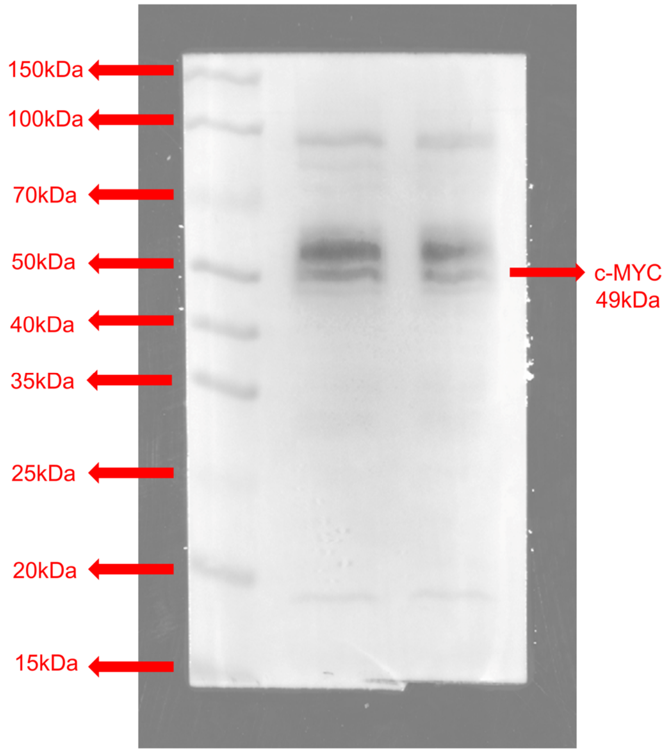

Figure 5 Fuller-length, original, and unprocessed Western blot images of Anti-c-MYC antibody to confirm specific detection of the target antigen. (Predicted band size: 49

kDa, Observed band size: 49 kDa)

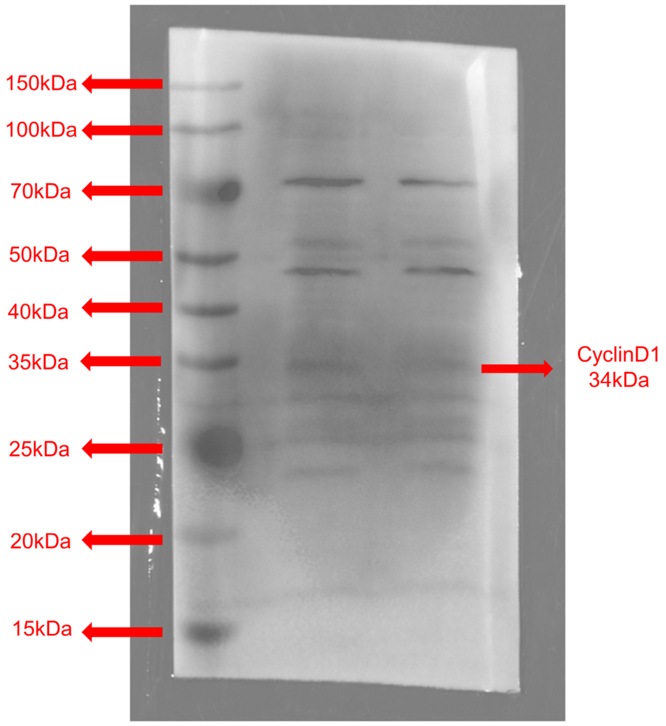

Figure 6 Fuller-length, original, and unprocessed Western blot images of Anti-CyclinD1 antibody to confirm specific detection of the target antigen. (Predicted band size: 34 kDa, Observed band size: 34 kDa)

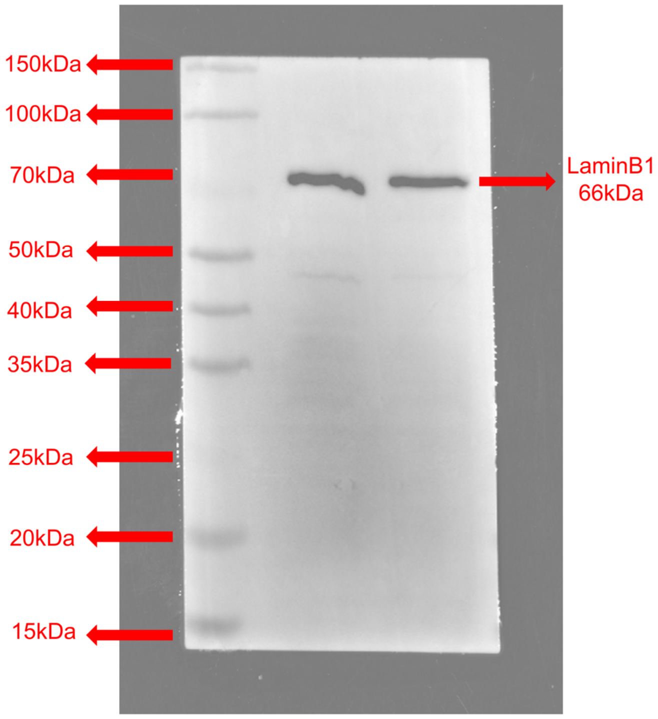

Figure 7 Fuller-length, original, and unprocessed Western blot images of Anti-LaminB1 antibody to confirm specific detection of the target antigen. (Predicted band size: 66 kDa, Observed band size: 66 kDa)

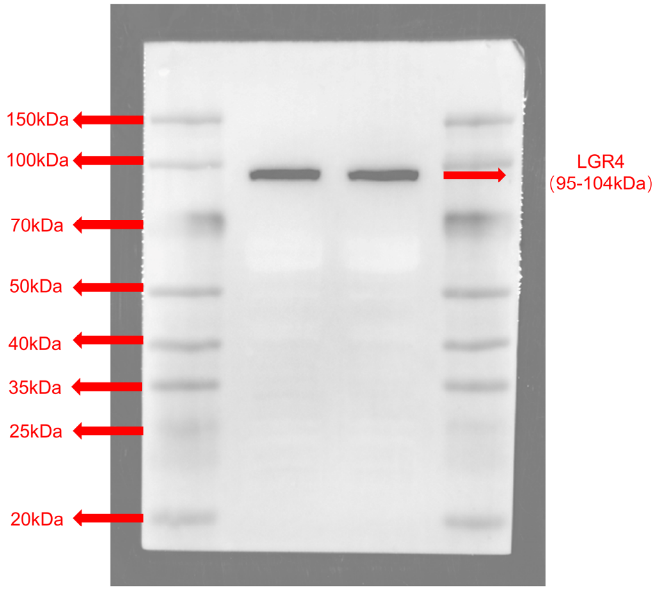

Figure 8 Fuller-length, original, and unprocessed Western blot images of Anti-LGR4 antibody to confirm specific detection of the target antigen. (Predicted band size: 104 kDa, Observed band size: 95-104 kDa)

.

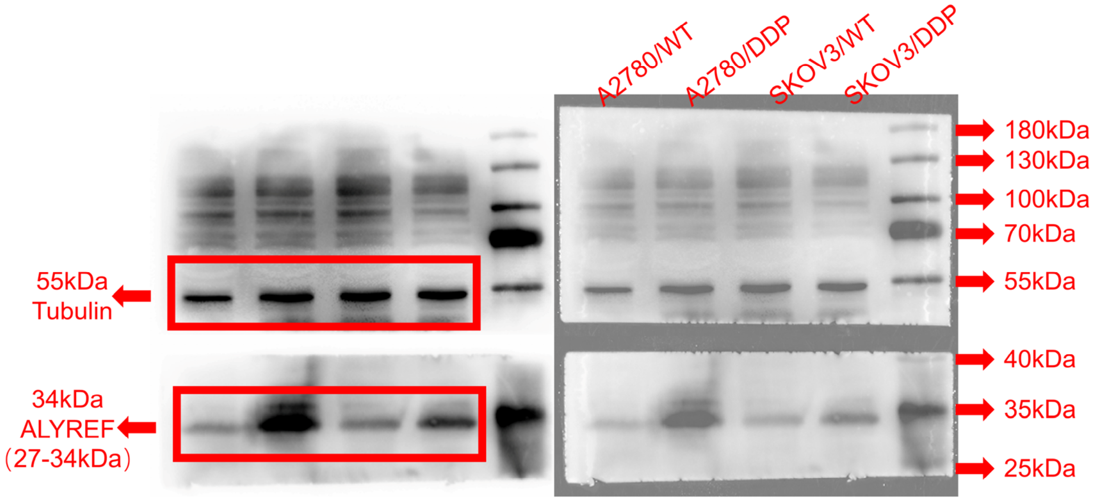

Figure 9 The original Western blot images in Figure 1E.
We cut between 40kDa -55kDa and incubated the upper half with anti-Tubulin antibody and the lower half with anti-ALYREF antibody.

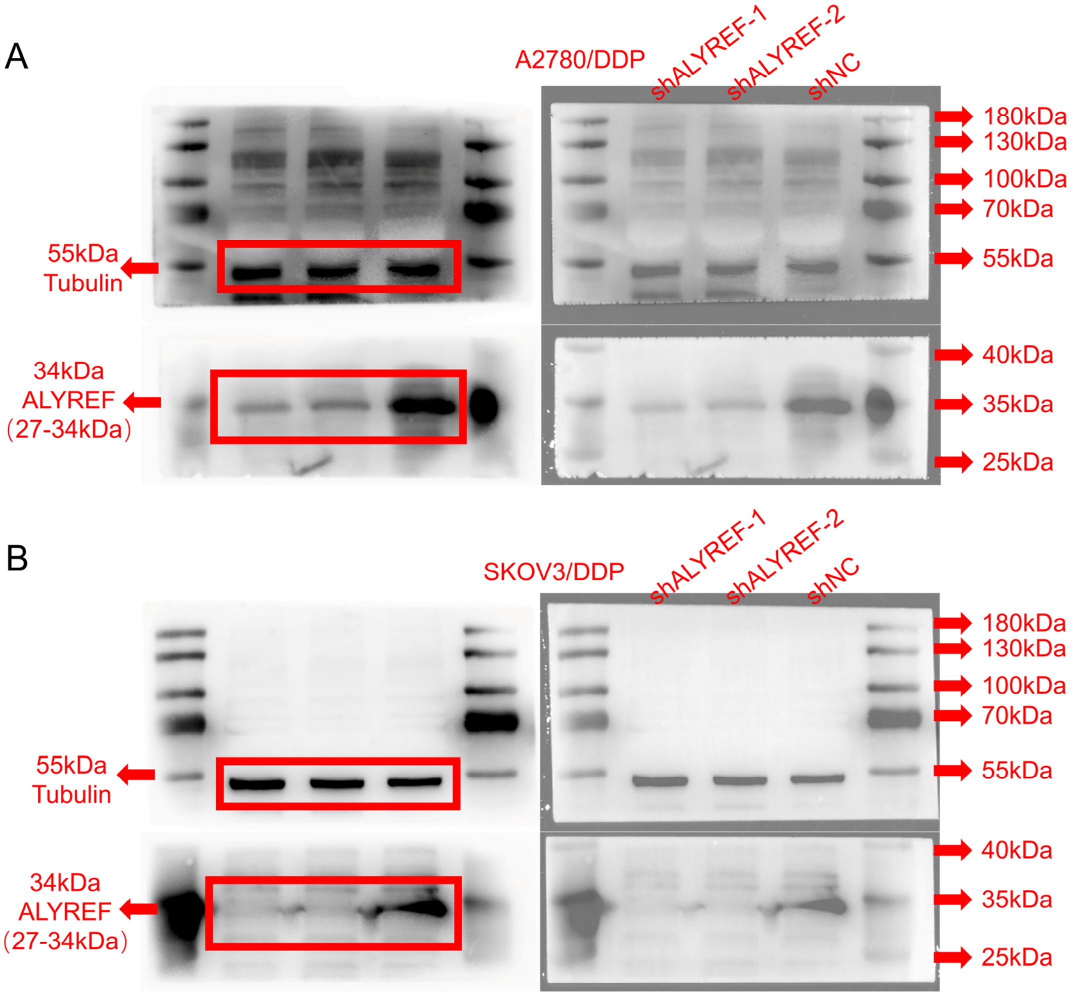

Figure 10 The original Western blot images of ALYREF knockdown in Figure 3A. (A) The original Western blot images of ALYREF knockdown in A2780/DDP in Figure 3A. (B) The original Western blot images of ALYREF knockdown in SKOV3/DDP in Figure 3A.

For Figure 3A, we cut between 40kDa -55kDa and incubated the upper half with anti-Tubulin antibody and the lower half with anti-ALYREF antibody.

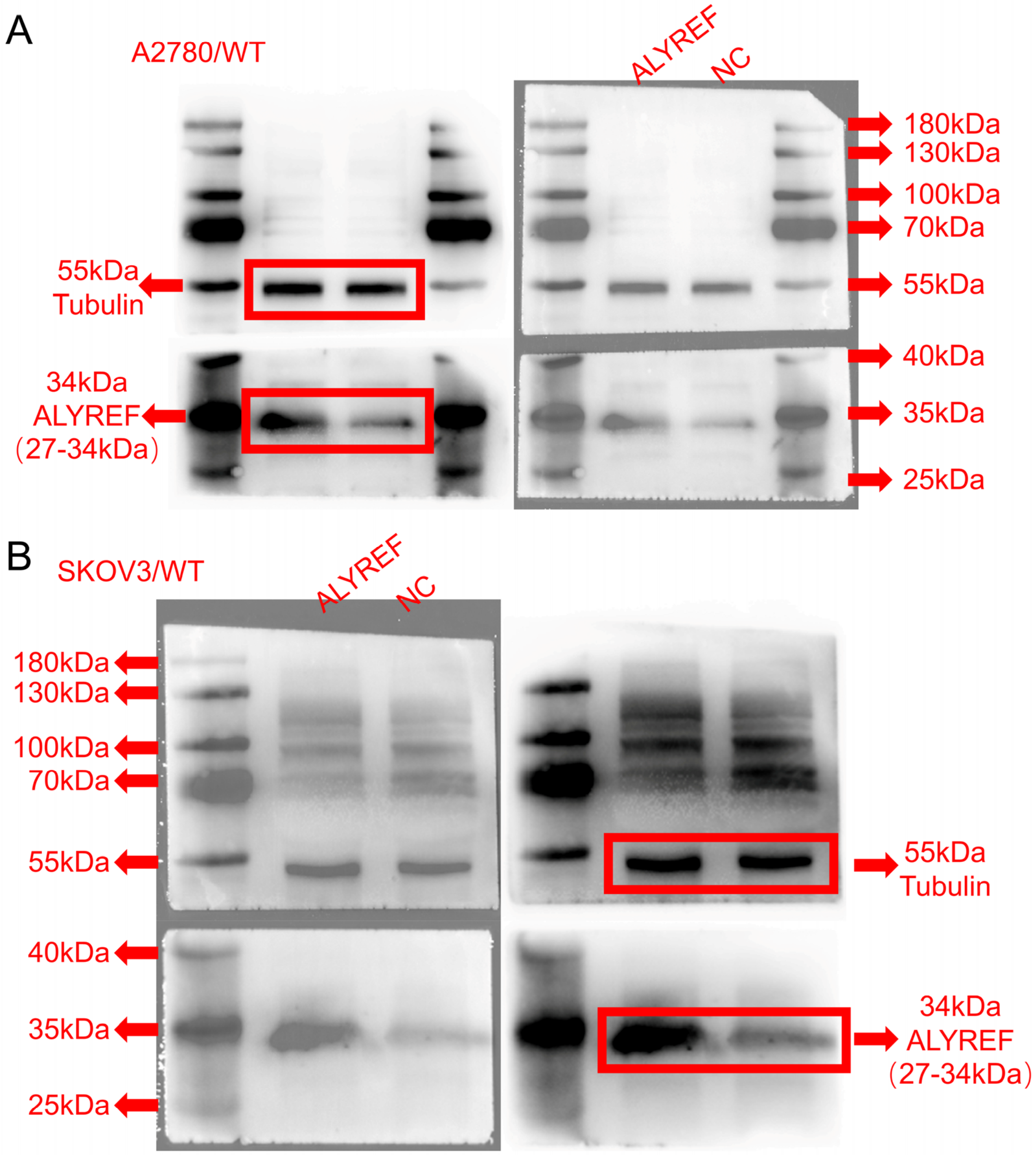

Figure 11 The original Western blot images of ALYREF overexpressing in Figure 3A. (A) The original Western blot images of ALYREF overexpressing in A2780/WT in Figure 3A. (B) The original Western blot images of ALYREF overexpressing in SKOV3/WT in Figure 3A.

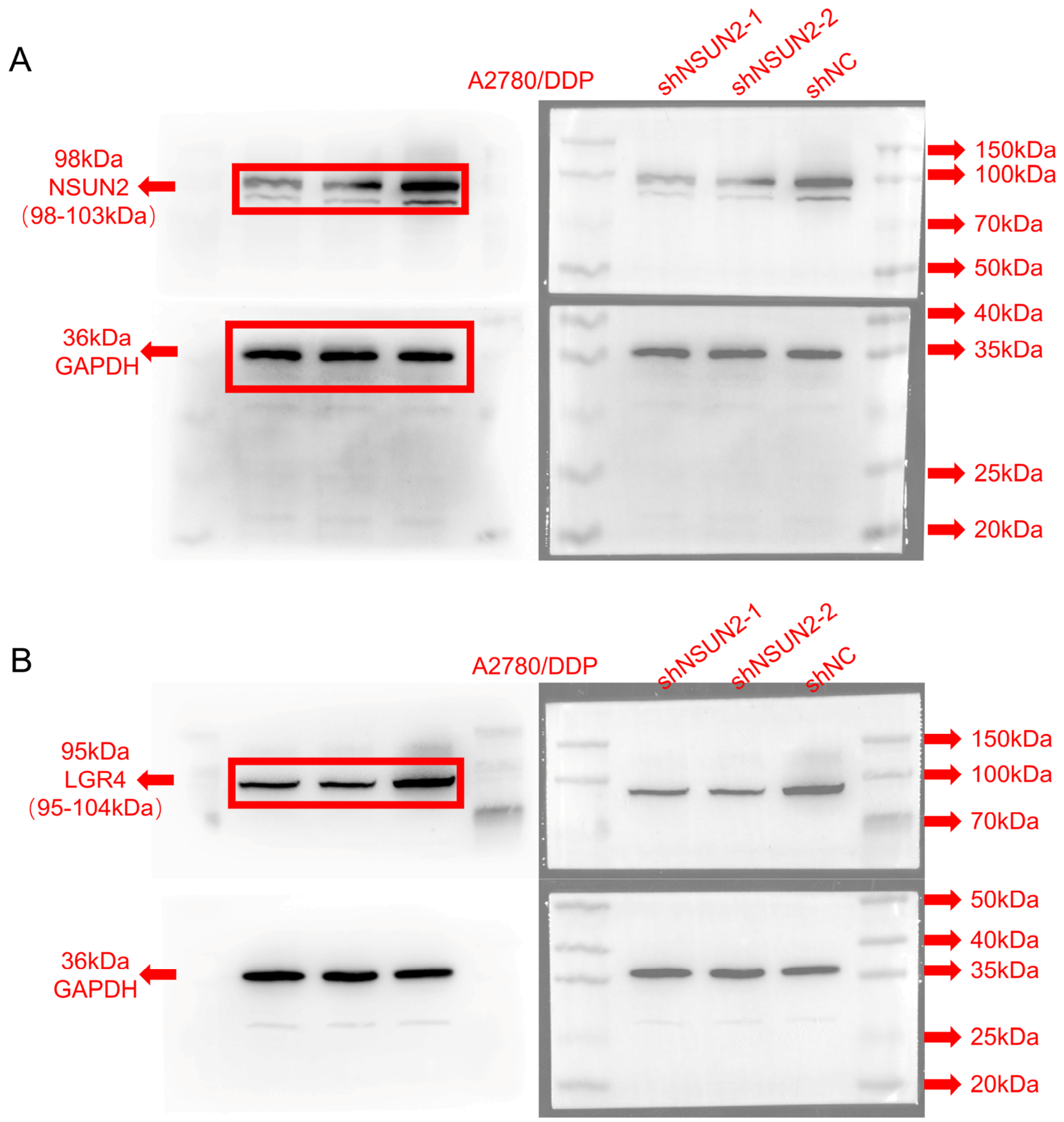

Figure 12 The original Western blot images of NSUN2 knockdown in A2780/DDP in Figure 5A.

(A) The original Western blot images of NSUN2 in NSUN2 knockout A2780/DDP cells.

(B) The original Western blot images of LGR4 in NSUN2 knockout A2780/DDP cells. For Figure 5A, we cut between 40kDa -50kDa and incubated the upper half with anti-NSUN2 antibody and the lower half with anti-GAPDH antibody. Additionally, we cut between 50kDa -70kDa and incubated the upper half with anti-LGR4 antibody and the lower half with anti-GAPDH antibody.

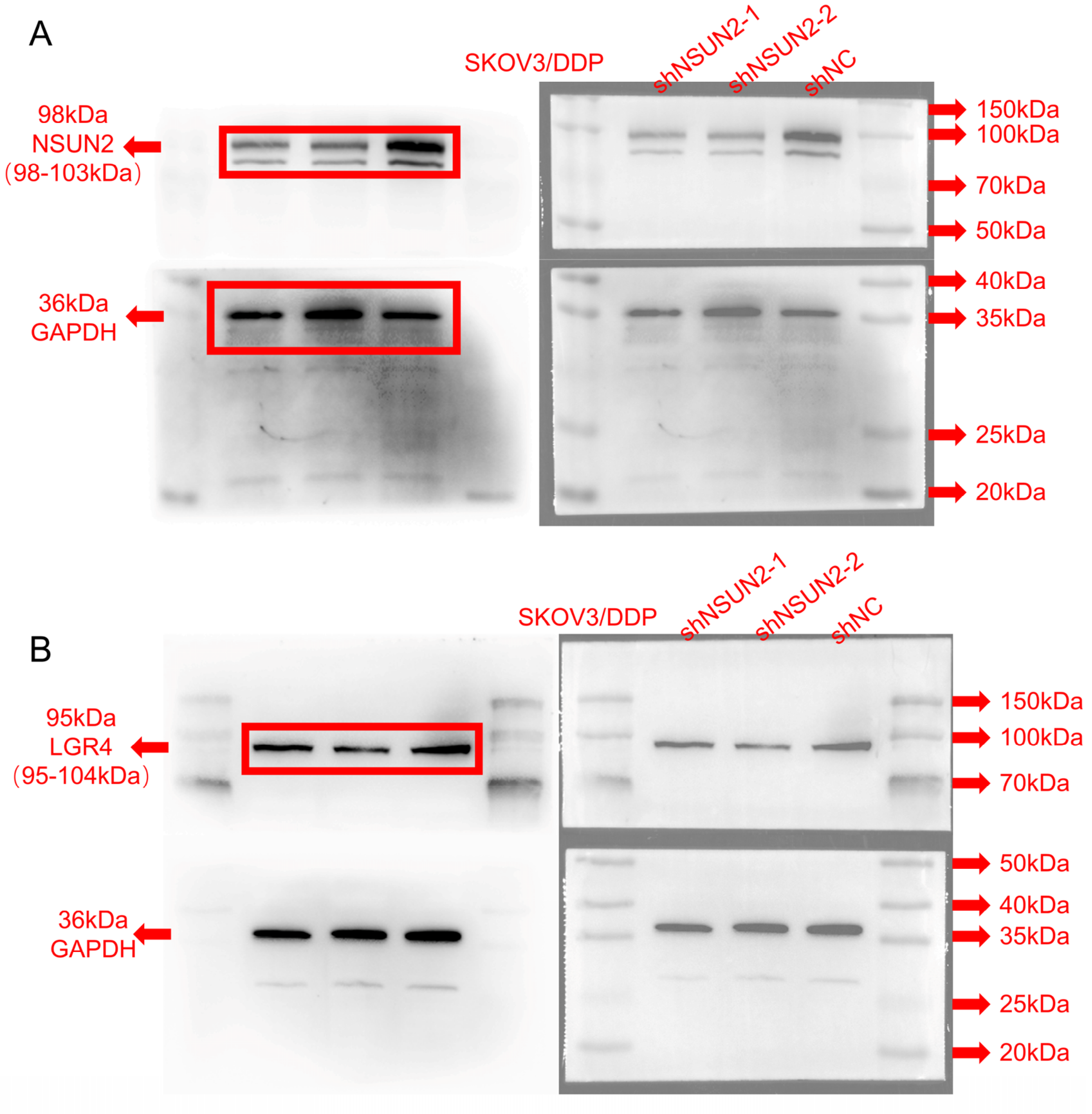

Figure 13 The original Western blot images of NSUN2 knockdown in SKOV3/DDP in Figure 5A.

(A) The original Western blot images of NSUN2 in NSUN2 knockout SKOV3/DDP cells. (B) The original Western blot images of LGR4 in NSUN2 knockout SKOV3/DDP cells.

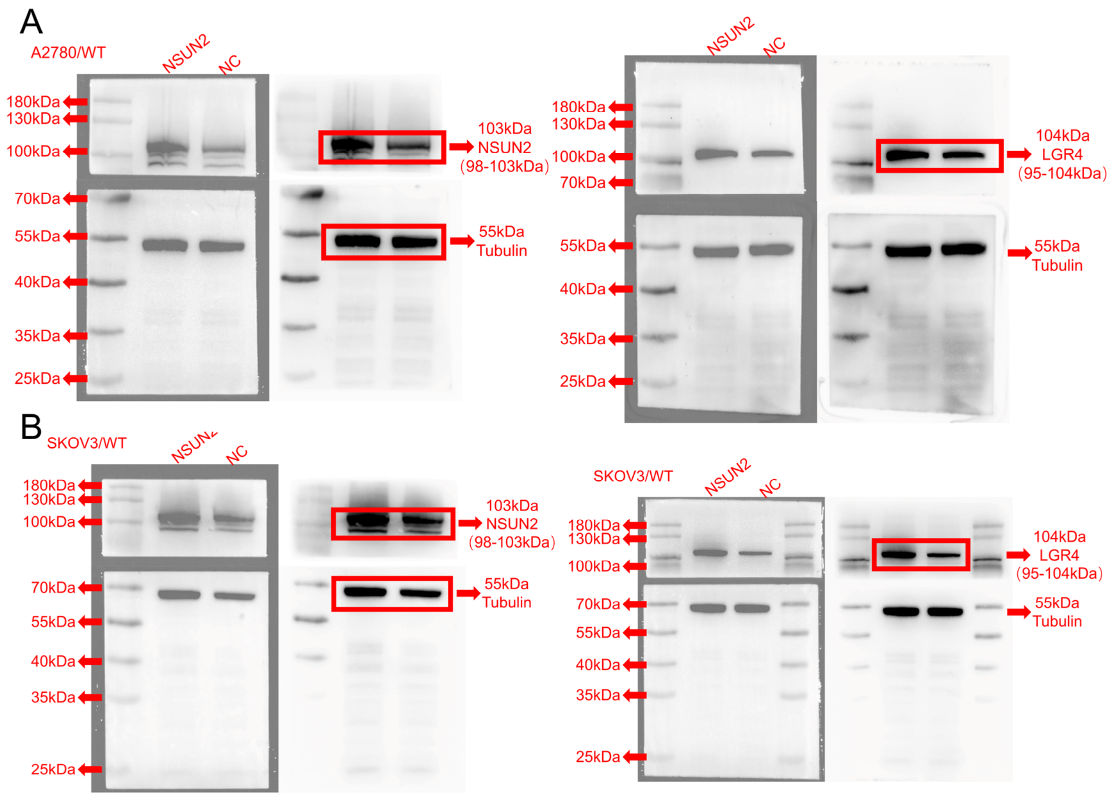

Figure 14 The original Western blot images of NSUN2 overexpressing in Figure 5A. (A) The original Western blot images of NSUN2 overexpressing in A2780/WT cells. (B) The original Western blot images of NSUN2 overexpressing in SKOV3/WT cells.

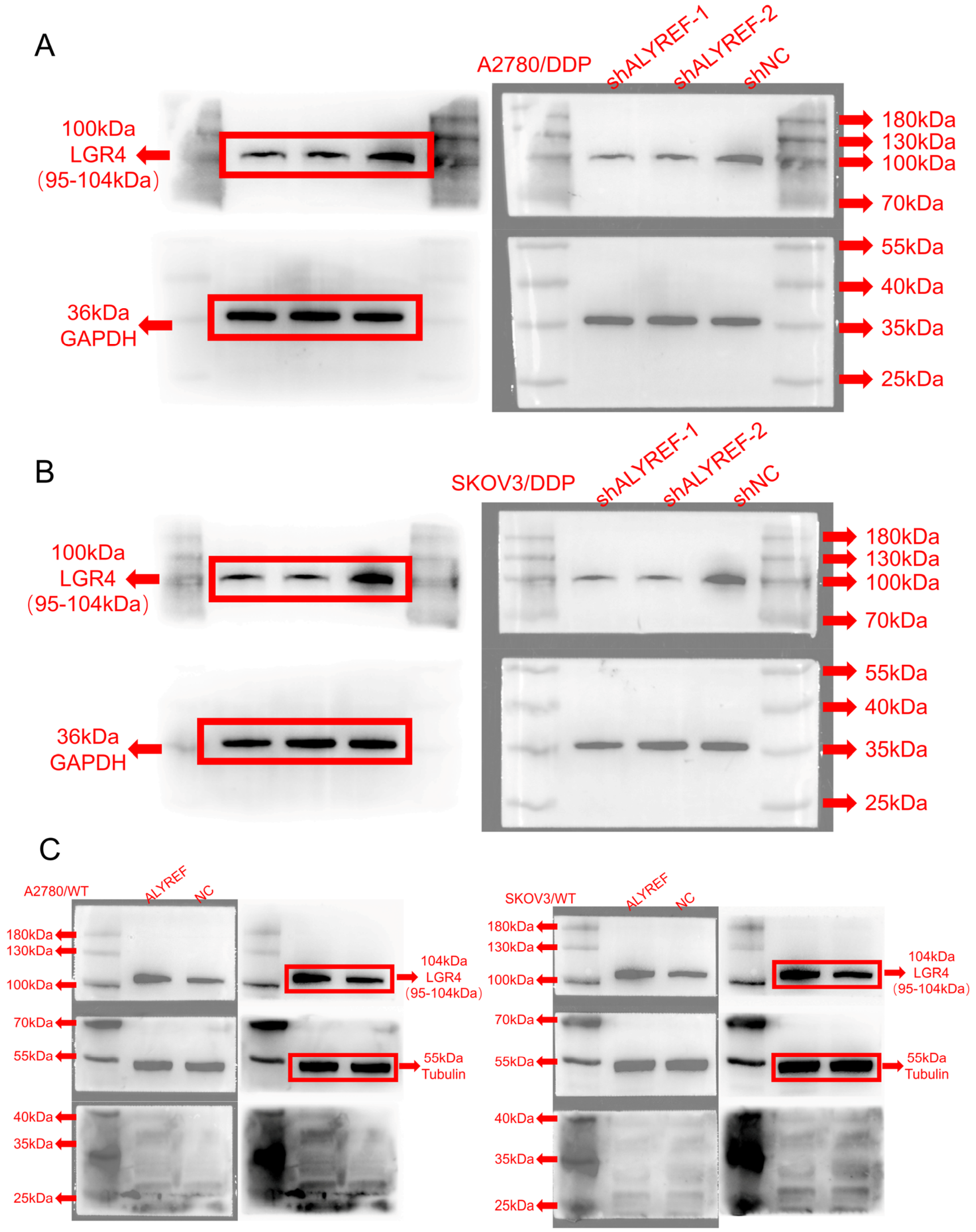

Figure 15 The original Western blot images in Figure 6C.

(A) The original Western blot images of ALYREF knockdown A2780/DDP cells in Figure 6C. (B) The original Western blot images of ALYREF knockdown SKOV3/DDP cells in Figure 6C.(C) The original Western blot images of ALYREF overexpressing in Figure 6C.

For the ALYREF knockdown cells in Figure 6C, we cut between 70-100kDa. The upper half was incubated with anti-LGR4 antibody, and the lower half was incubated with anti-GAPDH antibody. For the ALYREF-overexpressing cells in Figure 6C, we cut between 70-100kDa and between 40-55kDa. The bands were then incubated sequentially from top to bottom with anti-LGR4 antibody, anti-Tubulin antibody, and

anti-ALYREF antibody.

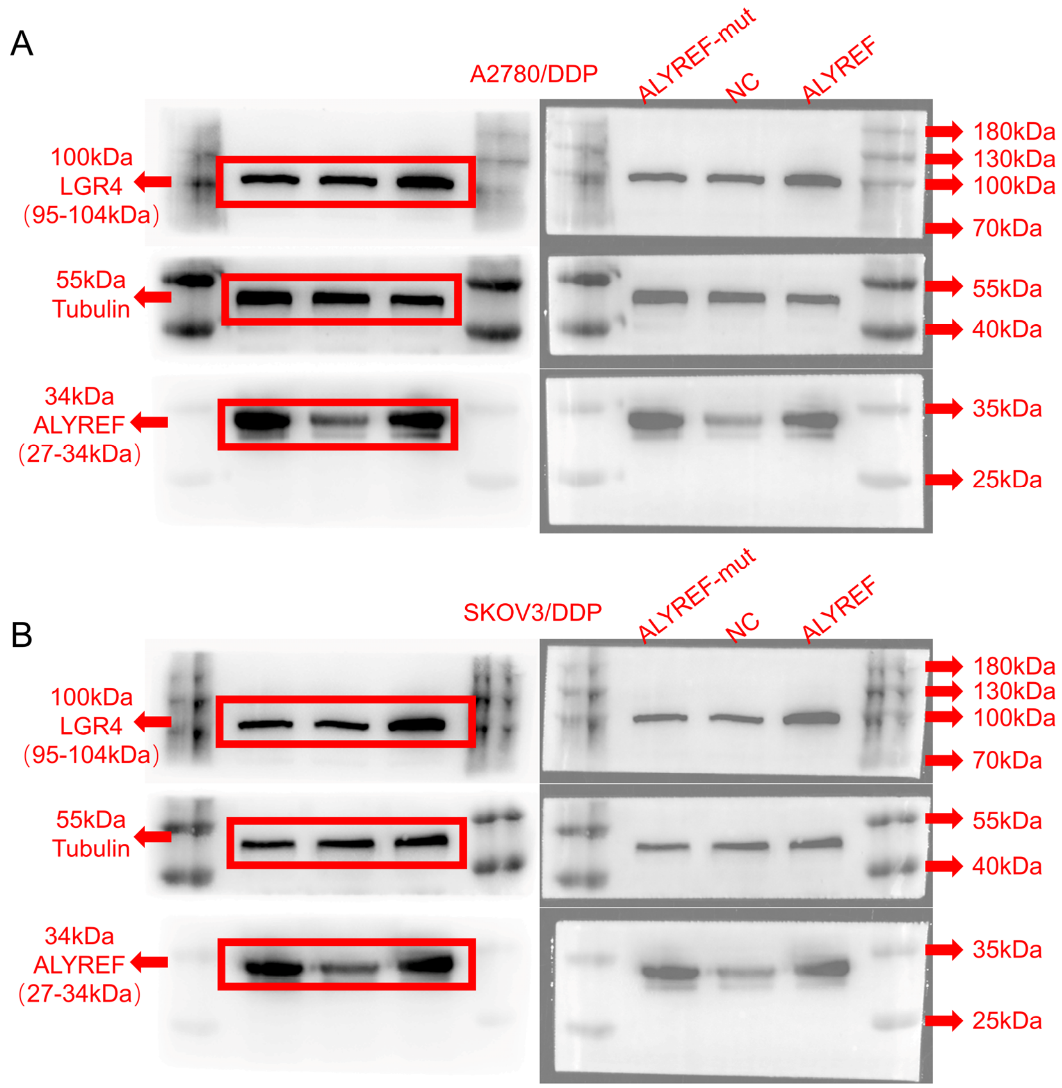

Figure 16 The original Western blot images in Figure 7D.
(A) The original Western blot images of ALYREF-mut A2780/DDP cells in Figure 7D.
(B) The original Western blot images of ALYREF-mut SKOV3/DDP cells in Figure 7D.
For Figure 7D, we cut between 55kDa-70kDa and between 35- 40kDa. The bands were then incubated sequentially from top to bottom with anti-LGR4 antibody, anti-Tubulin antibody, and anti-ALYREF antibody.

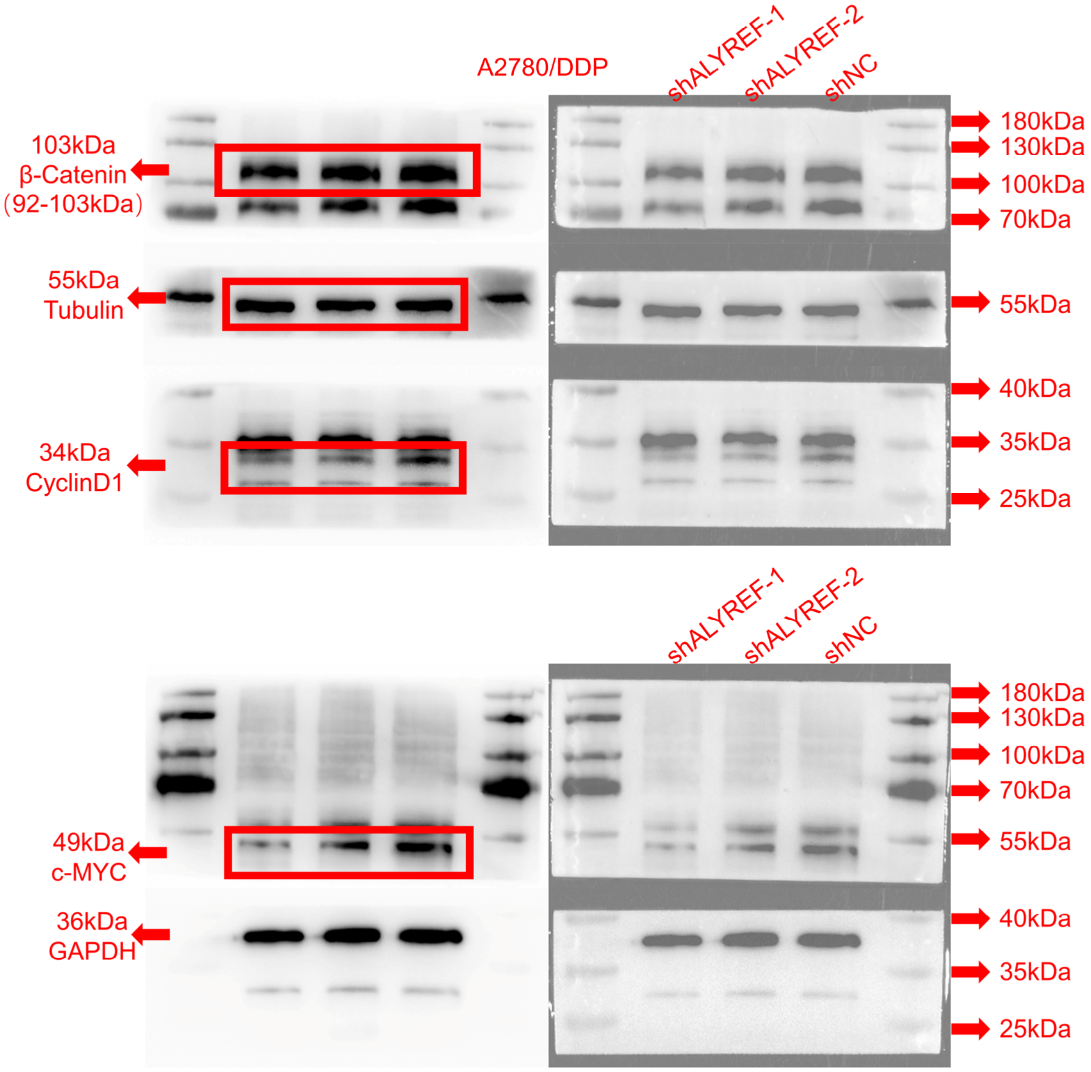

Figure 17 The original Western blot images of A2780/DDP in Figure S6A.

For Figure S6A, we cut between 55kDa-70kDa and between 40- 55kDa. The bands were then incubated sequentially from top to bottom with anti-β-Catenin antibody, anti-Tubulin antibody, and anti-CyclinD1 antibody. Additionally, we cut between 40kDa - 55kDa and incubated the upper half with anti-c-MYC antibody and the lower half with anti-GAPDH antibody.

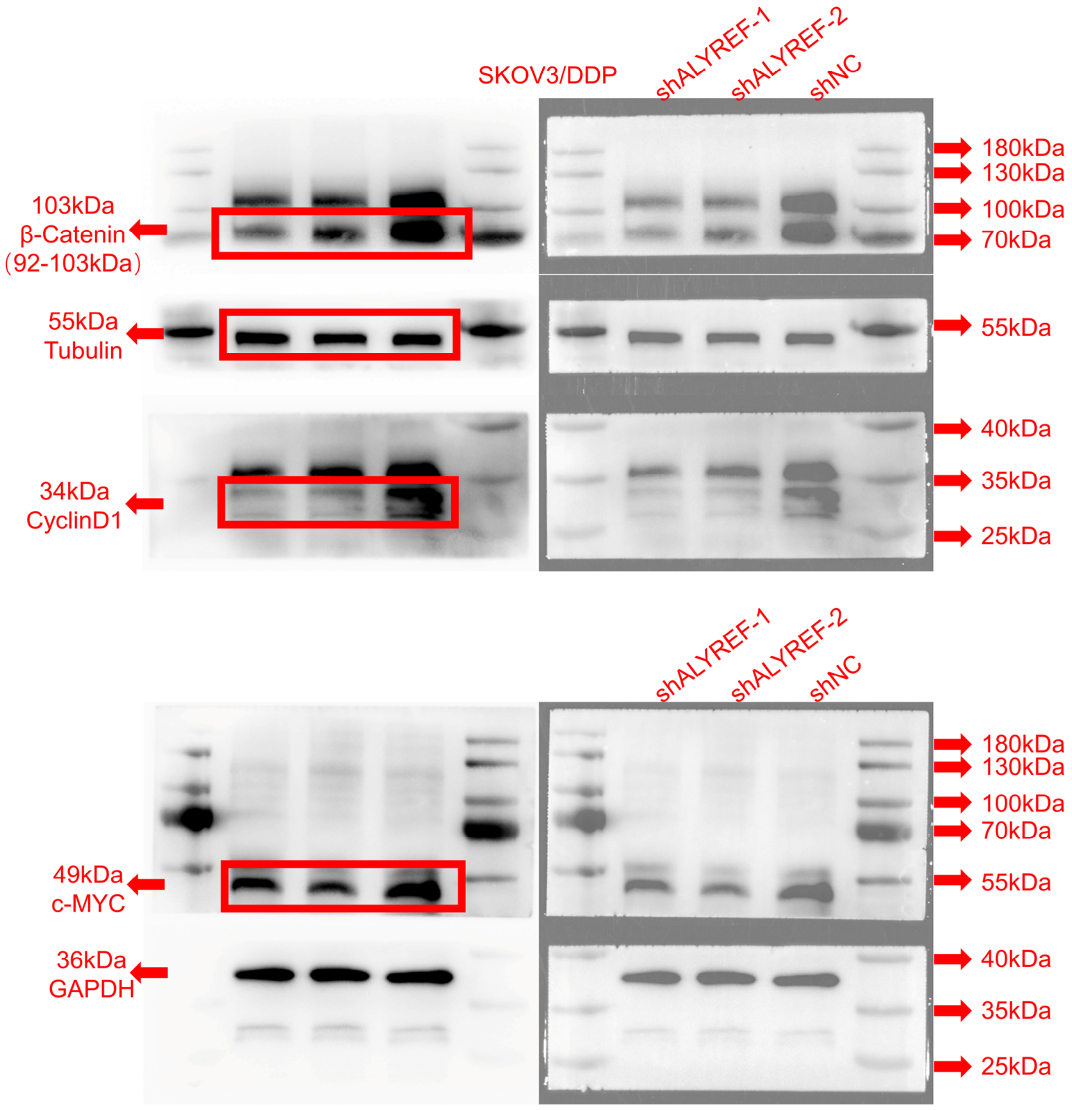

Figure 18 The original Western blot images of SKOV3/DDP in Figure S6A.

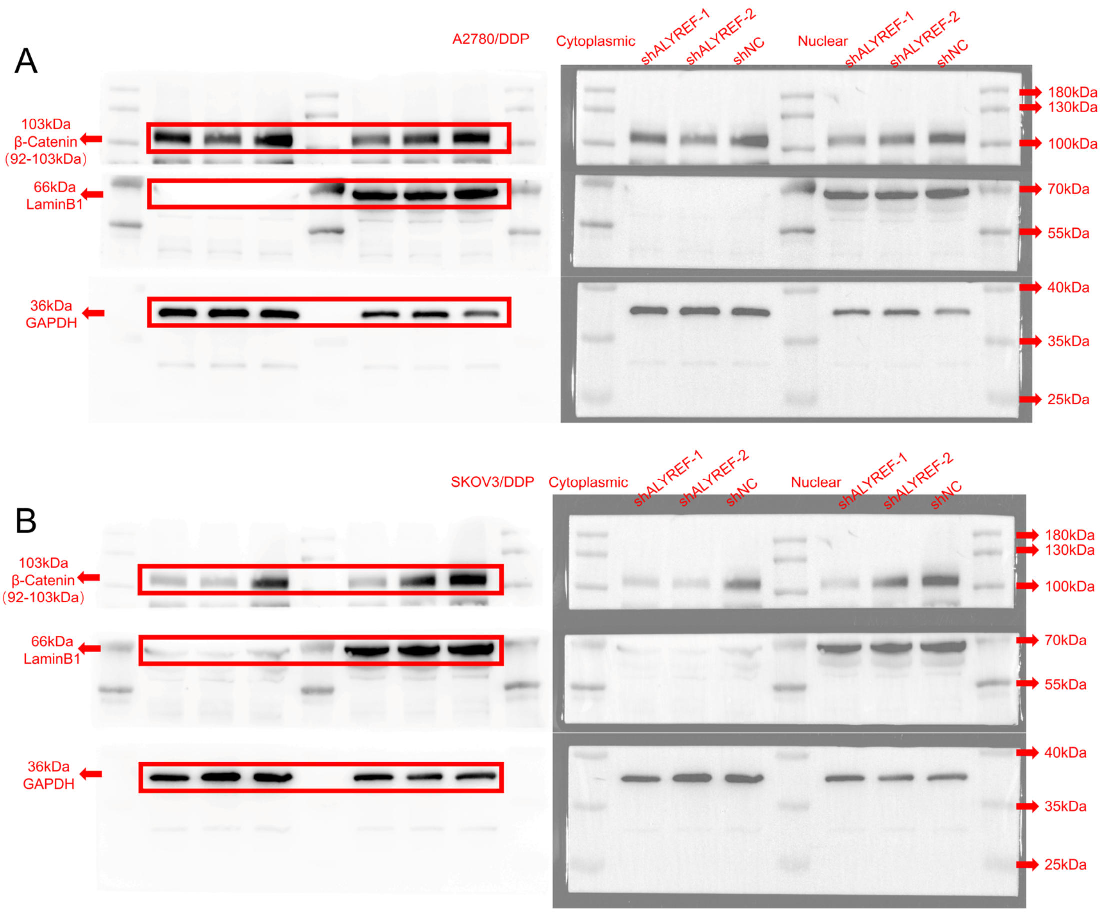

Figure 19 The original Western blot images in Figure S6B.

For Figure S6B, we cut between 70-100kDa and between 40-55kDa. The bands were then incubated sequentially from top to bottom with anti-β-Catenin antibody, anti-LaminB1 antibody, and anti-GAPDH antibody.

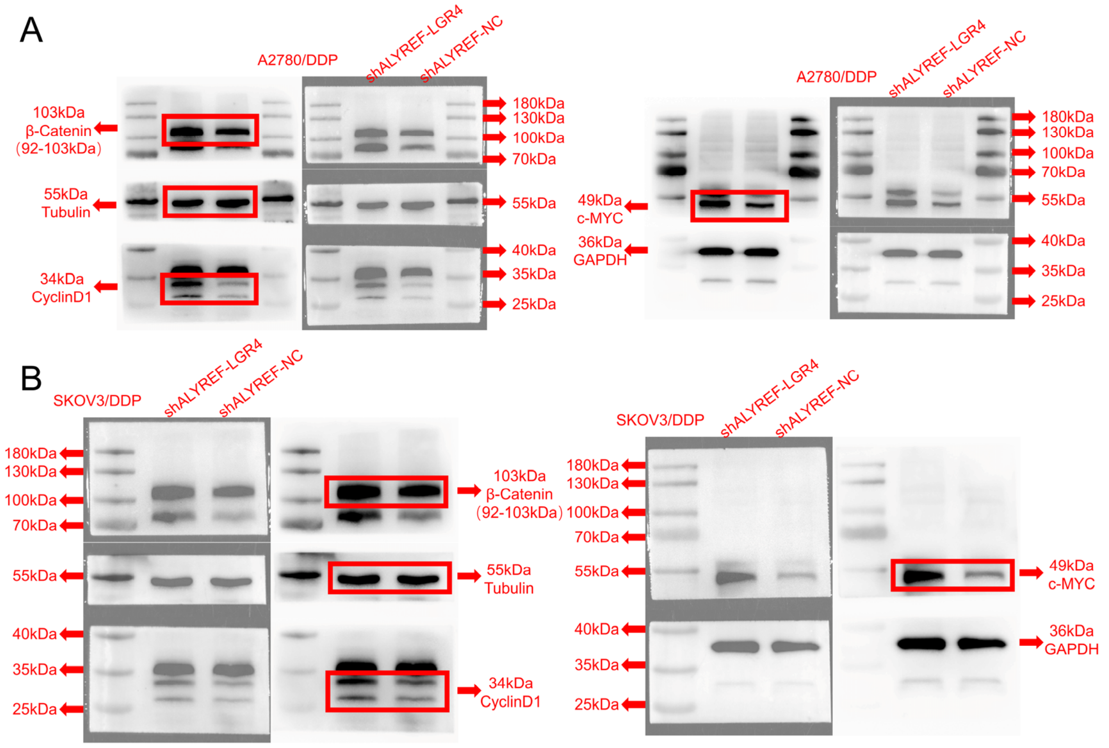

Figure 20 The original Western blot images in Figure S8F.

For Figure S8F, we cut between 55-70kDa and between 40-55kDa. The bands were then incubated sequentially from top to bottom with anti-β-Catenin antibody, anti-Tubulin antibody, and anti-CyclinD1 antibody. Additionally, we cut between 40-55kDa and incubated the upper half with anti-c-MYC antibody and the lower half with anti-GAPDH antibody.
